# The Effect of Covalently-Attached ATRP-Synthesized Polymers on Membrane Stability and Cytoprotection in Human Erythrocytes

**DOI:** 10.1371/journal.pone.0157641

**Published:** 2016-06-22

**Authors:** William P. Clafshenkel, Hironobu Murata, Jill Andersen, Yehuda Creeger, Richard R. Koepsel, Alan J. Russell

**Affiliations:** 1 The Institute for Complex Engineered Systems, Carnegie Mellon University, Pittsburgh, Pennsylvania, United States of America; 2 Molecular Biosensor and Imaging Center, Carnegie Mellon University, Pittsburgh, Pennsylvania, United States of America; Chang Gung University, TAIWAN

## Abstract

Erythrocytes have been described as advantageous drug delivery vehicles. In order to ensure an adequate circulation half-life, erythrocytes may benefit from protective enhancements that maintain membrane integrity and neutralize oxidative damage of membrane proteins that otherwise facilitate their premature clearance from circulation. Surface modification of erythrocytes using rationally designed polymers, synthesized via atom-transfer radical polymerization (ATRP), may further expand the field of membrane-engineered red blood cells. This study describes the fate of ATRP-synthesized polymers that were covalently attached to human erythrocytes as well as the effect of membrane engineering on cell stability under physiological and oxidative conditions *in vitro*. The biocompatible, membrane-reactive polymers were homogenously retained on the periphery of modified erythrocytes for at least 24 hours. Membrane engineering stabilized the erythrocyte membrane and effectively neutralized oxidative species, even in the absence of free-radical scavenger-containing polymers. The targeted functionalization of Band 3 protein by NHS-pDMAA-Cy3 polymers stabilized its monomeric form preventing aggregation in the presence of the crosslinking reagent, bis(sulfosuccinimidyl)suberate (BS_3_). A free radical scavenging polymer, NHS-pDMAA-TEMPO˙, provided additional protection of surface modified erythrocytes in an *in vitro* model of oxidative stress. Preserving or augmenting cytoprotective mechanisms that extend circulation half-life is an important consideration for the use of red blood cells for drug delivery in various pathologies, as they are likely to encounter areas of imbalanced oxidative stress as they circuit the vascular system.

## Introduction

Both intact and hemoglobin-free erythrocyte ghosts have been suggested as advantageous biological vehicles for therapeutic delivery [1]. However, the utility of red blood cells (RBCs) as drug delivery systems is dependent on their half-life in circulation, which can be greatly altered by events that prematurely activate clearance mechanisms. Although erythrocytes are equipped with effective antioxidant systems that offer protection to themselves and other tissues and organs in the body [2], a weakening of this defense system allows for the accumulation of oxidatively damaged lipids and proteins, including hemoglobin and Band 3 [3]. The proteolytic modification or aggregation of Band 3 in the erythrocyte membrane can be induced by oxidant exposure, oxidized and denatured hemoglobin, hyperphosphorylation by src kinases, poor glycosylation, and crosslinking agents such as stilbenes [4–6].

There are several mechanisms by which antioxidants prevent oxidative damage, including the interception of free radicals by scavenging the reactive metabolites and converting them to less reactive molecules [7]. However, if the antioxidant defense system is overpowered by excessive production of free radicals or if there is a decrease in the level of antioxidants, the resulting imbalance will lead to oxidative stress with deleterious effects. Oxidative stress has been correlated to the progression of acute and chronic pathologies such as inflammatory conditions, cancer, and cardiovascular disease [8, 9]. Reactive oxygen species (ROS) mediate oxidative damage to cellular proteins, lipids, and DNA via highly reactive unpaired electrons [10, 11]. In particular disorders like inflammatory diseases and cancer, oxidative stress is markedly increased and ROS are more abundant [12, 13]. Thus, there may be great impetus for scavenging reactive chemical species when developing targeted biomedical therapeutics, specifically those aimed at treating inflammatory conditions and cancer.

Phagocytosis of damaged red blood cells by macrophages is facilitated by the binding of naturally occurring autoantibodies (NAbs) to membrane proteins like Band 3 and spectrin [14]. Additionally, phosphatidylserine (PS) becomes more numerous in the outer membrane leaflet of erythrocytes as they age, are subject to oxidative stress, and undergo lipid peroxidation [14–16]. The externalization of PS on the cell surface has been identified as an early and prominent feature of apoptosis, promoting the cell’s recognition and removal by phagocytes [17]. Macrophages recognize externalized PS through several receptors, including PS receptors, Tim1, Tim4, and Stabilin-2 [18].

Several studies have demonstrated that an increase in circulation half-life can be achieved by covalently modifying the surface of erythrocytes with polymers such as poly(ethylene) glycol (PEG) or methoxy-PEG (mPEG). Surface polymer coating masks ABO and Rh antigenic sites that would otherwise provide detection by immune surveillance [1, 19, 20]. Murine RBCs modified with mPEG and injected back into donor mice showed normal *in vivo* survival of approximately fifty days [19]. Interestingly, sheep RBCs modified with mPEG and injected into mice had a significantly longer half-life in circulation compared to unmodified sheep RBCs [20]. Additionally, erythrocytes decorated with PEG demonstrated reduced aggregation and reduced low shear blood viscosity [21]. Despite the advances these studies have made in exploring “stealth” erythrocytes, whether the surface modification of erythrocytes with polymers has a significant role in mitigating clearance mechanisms related to the accumulation of oxidative stress or membrane stabilization remains unexplored. These pioneering studies on the membrane engineering of red blood cells are, however, under increased scrutiny because of the emergence of significant immune responses to PEG itself. Alternative polymers which have the benefits of PEG without the drawbacks are essential to develop and assess.

The functionalization of fullerene derivatives [8] and hemoglobin-based oxygen carriers [22] with free radical-scavenging moieties has been cited to be valuable to their function as *in vivo* cytoprotective therapeutic agents. Moreover, endogenous and/or exogenous reducing agents such as ascorbic acid can suppress hemoglobin oxidation both *in vitro* and *in vivo*, and erythrocytes are protected against oxidative stress by vitamin E, glutathione, N-acetylcysteine, and other antioxidants [15, 23]. Interestingly, the cell-permeable, stable nitroxide radical TEMPO˙ (2,2,6,6-tetramethylpiperidin-1-yl)oxyl) and its hydroxylated form, Tempol (4-hydroxy-TEMPO), have illustrated significant antioxidant effects [24–27]. Both protect cells from inducers of oxidative damage such as superoxide, hydrogen peroxide, and ionizing radiation [27]. Their antioxidant behavior is likely driven by their activity as superoxide dismutase and/or catalase mimics, the reduction in the formation of hydroxyl radicals, and radical-radical interactions [27]. The reaction of free radical scavenging polymers with proteins has also been utilized to stabilize enzymes against photodegradation induced by TiO_2_-UV exposure [28]. These studies suggest that nitroxides or other chemical species could potently mitigate oxidative reactions that impact red blood cell viability and clearance.

Rather than modify naked hemoglobin, which unnecessarily removes the protein from its protective shell and sophisticated enzymatic and nonenzymatic antioxidant defense systems, we have begun to explore erythrocyte cytoprotective mechanisms using surface modification of intact erythrocytes with non-PEG polymers (pDMAA), including a polymeric TEMPO derivative. The capacity to neutralize oxidative species is an important consideration for the use of cell-based drug delivery methods that are likely to encounter areas of imbalanced oxidative stress that would compromise therapeutic efficacy. There are several advantages to using ATRP to synthesize biofunctional polymers. Indeed, our group has demonstrated the utility of these polymers in tailoring and stabilizing enzyme activity [29], for the targeting of mesenchymal stem cells to bone tissue [30], and for regulating biological interactions [31]. Additionally, polymer chain length and substitution is easily controlled under mild reaction conditions, and the synthesized polymers are separated from the reaction mixture without expensive or complex separation techniques [32].

Prior to determining the impact of membrane engineering in a model of erythrocyte oxidative stress, the surface density and retention of N-hydroxysuccinimide (NHS) functionalized pDMAA polymers on the erythrocyte surface and the effect of polymer chains on membrane destabilization were evaluated. We also determined the impact of membrane engineering on the distribution of Band 3 protein, a major integral protein that participates in the *in vivo* clearance of erythrocytes [14]. Additionally, epifluorescence microscopy and flow cytometry were utilized to determine the degree of protection afforded to erythrocytes modified with and without TEMPO-containing polymer chains by monitoring the externalization of phosphatidylserine, an event synonymous with erythrocyte membrane oxidation [15, 16].

## Materials and Methods

### Erythrocytes

Packed, leukocyte-reduced, Rh-positive human erythrocytes (hRBCs) were obtained from healthy volunteer donors through the Central Blood Bank in partnership with the Institute of Transfusion Medicine (Pittsburgh, PA). Utilization of blood products for the experiments conducted was approved by the Carnegie Mellon University Institutional Review Board and followed all protocols for the processing and handling of such samples. Packed red blood cells were stored in a gas-permeable unit bag at 4°C until use or expiration (approximately 42 days after collection).

### Exposure of intact and ghost human erythrocytes (hRBC) to membrane-reactive polymers

Human red blood cells were exposed to either a rhodamine (Rh) or cyanine 3 (Cy3)-tagged non-binding control polymer chain (HO-pDMAA-Rh and HO-pDMAA-Cy3, respectively), a dye-tagged NHS-functionalized polymer chain (NHS-pDMAA-Rh or NHS-pDMAA-Cy3), or an untagged NHS-pDMAA-TEMPO˙ polymer chain by adapting methods previously described by our group [30]. Fresh stock solutions of lyophilized polymer were completely dissolved in isotonic PBS, pH 7.4 (1X PBS) or DMSO. Initial experiments with rhodamine demonstrated high non-specific binding of polymers to the erythrocyte surface as discussed below. Therefore, the utility of an alternate dye moiety, cyanine 3, for polymer localization studies was investigated. A disulfo-Cy3 fluorescent label was chosen to increase solubility over underivatized forms. Briefly, 1×10^8^ hRBC/mL were exposed to polymer by gently rocking in a HO- and NHS-pDMAA-Cy3 (100 μM), NHS-pDMAA-Rh (182 μM), or HO-pDMAA-Rh (96 μM) polymer solution for 30 minutes at 37°C. After incubation, hRBCs were spun at 16 K rcf and washed three times with 1X PBS to remove residual, unbound polymer. Modified hRBCs were used immediately for experiments and exposure to light was limited during analyses.

For the modification of ghost erythrocytes, hRBC ghost cells were created by hypotonic lysis [33, 34]. Briefly, 1×10^8^ hRBC/mL were lysed by adding ice-cold deionized water to packed cells, pipetting vigorously, and then incubating on ice for 5 minutes. Afterwards, hRBC ghosts were pelleted by centrifugation at 16 K rcf at 4°C for 5 minutes. The cytosolic fraction containing release hemoglobin was carefully removed. The light pink to milky white pellet was resuspended in ice-cold deionized water, incubated on ice for 5 minutes, and then respun at 16 K rcf at 4°C for 5 minutes. The pelleted ghosts were resuspended and modified by the methods described above for intact cells. Aliquots of modified ghost hRBCs were imaged at 0, 1, and 2 hours using a Leica DM IRB inverted microscope fitted with a TRITC filter cube (Leica Microsystems GmbH, Wetzlar, Germany). The resulting fluorescent images were background corrected and color balanced using Image J software. Information regarding the molecular characteristics of the synthesized polymers, including their average molecular weight and polydispersity index, can be found in the S1 File.

### The fate of polymers reacted with the membranes of hRBCs

Human red blood cells were modified as described above. Following modification, hRBCs were incubated for 0, 1, 4, 8 or 24 hrs at 37°C with gentle rocking in 1X PBS. At each time point, hRBCs were spun at 16 K rcf for 3 minutes. The supernatant was then removed. Pelleted hRBCs were lysed hypotonically by vigorous pipetting after the addition of ice-cold deionized water. The hRBCs were vortexed and kept on ice for 5 minutes before centrifugation at 16 K rcf at 4°C for 5 minutes. After centrifugation, the supernatant (cytosolic fraction) was collected, leaving behind hRBC ghosts, which were resuspended in PBS. All collected fractions were kept refrigerated until analysis. Aliquots of intact hRBCs for each time point were imaged using a Leica DM IRB inverted microscope fitted with a TRITC filter cube (Leica Microsystems GmbH, Wetzlar, Germany). The resulting fluorescent images were background corrected and color balanced using Image J software.

Polymer concentration in each fraction was determined by measuring the relative fluorescent intensity of rhodamine or Cy3 using a Synergy H1 Hybrid multi-mode microplate reader (BioTek, Winooski, VT). The number of polymer molecules per hRBC and the number of molecules per nm^2^ of hRBC surface area were calculated based on a standard curve generated from serial dilutions of polymer and an average RBC surface area of 140 μm^2^, respectively [35].

### The impact of membrane engineering on Band 3 protein aggregation under physiological conditions

The aggregation of Band 3 membrane protein in hRBCs was assessed by determining the membrane content of Band 3 using SDS-PAGE. Additionally, both polymer-modified and unmodified hRBCs were exposed to the membrane-impermeable, protein crosslinking agent bis(sulfosuccinimidyl)suberate (BS_3_) to investigate the effect of polymer modification on the stabilization of monomeric Band 3. Human erythrocytes were modified with NHS-pDMAA-Cy3 polymer chains as described above. After the final wash, hRBCs were pelleted at 16 K rcf for 5 minutes at 4°C. The intact hRBC were lysed in ice-cold 5 mM sodium phosphate buffer, pH 8.0 containing protease inhibitors and 1 mM EDTA (Complete Mini Tablets, Roche Applied Science, Indianapolis, IN). Ghost hRBCs were pelleted by centrifugation at 16 K rcf for 30 minutes at 4°C, washed once with lysis buffer, and respun. The protein content of ghost membranes was determined by a modified Lowry protein assay (Thermo Scientific Pierce, Rockford, IL).

In order to determine polymer interaction with individual membrane-associated proteins in addition to Band 3, such as Band 4.1, Band 4.2, and glycophorin A, the samples were run under reducing conditions. Samples were diluted to equivalent protein content (approximately 8 μg/μL) and solubilized with gel loading buffer containing 1% fresh β-mercaptoethanol, 2% SDS, 15% v/v glycerol, and 0.02% bromophenol blue in 50 mM Tris HCl, pH 6.8. Samples were then loaded onto a 12% mini-Protean TGX precast gel and subjected to SDS-PAGE according to the methods of Laemmli [36] at 110V for approximately one hour. The resulting protein bands were stained with Imperial protein stain (Thermo Scientific Pierce, Rockford, IL) per the manufacturer’s instructions. Rinsed gels were digitally scanned and apparent molecular masses of erythrocyte proteins were approximated based on the band migration of a molecular weight standard (Precision Plus Kaleidoscope protein standards, Bio-Rad, Hercules, CA). Additional gel analysis was carried out using Image J software.

### Impact of membrane engineering on the membrane stability of hRBCs under physiological and oxidizing conditions

Membrane destabilization in polymer-modified hRBCs under physiological and oxidizing conditions was assessed by monitoring the translocation of phosphatidylserine (PS) from the inner membrane leaflet to the outer membrane leaflet using a commercially available kit (Dead Cell Apoptosis Kit, Life Technologies, Grand Island, NY). Phosphatidylserine (PS) becomes more numerous in the outer membrane leaflet of erythrocytes as they age, are subject to oxidative stress, and undergo lipid peroxidation [14–16] making it a reliable marker of membrane damage. Annexin V binds with high affinity to PS in a calcium-dependent manner, thus fluorescently tagged Annexin V in the kit can be used to identify hRBCs with exposed PS. Unmodified hRBC oxidized with 0.2 mM CuSO_4_/2.5 mM L-ascorbic acid were used as a positive control for Annexin V-Alexa 488 binding. Unmodified hRBCs in 1X PBS were used as a negative control. Annexin V-Alexa 488 binding was imaged in polymer-modified hRBCs under physiological conditions (incubation in isotonic PBS, pH 7.4) and under oxidizing conditions (incubation in 0.2 mM CuSO_4_/2.5 mM L-ascorbic acid) at 10, 30, 60, and 120 minutes. Images of Cy3 and Annexin V-Alexa 488 fluorescence were captured from each experimental group using a Leica DM IRB inverted microscope (Leica Microsystems, GmbH, Wetzlar, Germany). Individual channels for Cy3 and Annexin V-Alexa 488 were background-corrected, color-balanced, and merged using Image J software.

For flow cytometry, unmodified and polymer-modified hRBCs were incubated in 0.1 mM CuSO_4_/1.25 mM L-ascorbic acid at 10, 30, 60, and 120 minutes. PS externalization was detected as outlined above. After Annexin V-Alexa 488 binding, hRBC were washed once and distributed into individual wells of a 96-well plate in triplicate (1,000 hRBC/μL). The evaluation of Annexin V-Alexa 488 intensity was completed on an Accuri C6 flow cytometer (BD Biosciences, San Jose, CA) connected to an Intellicyt HyperCyt autosampler (IntelliCyt Corp., Albuquerque, NM) with Intellicyt ForeCyt v.3.1 software. The flow cytometer was equipped with a 488 and 635 laser and the emission filter used was a 533/30. Data were processed and interpreted using FlowJo software.

A recent publication has demonstrated that some cyanine dyes can be potent free-radical scavengers [37]. Additionally, both cyanine and rhodamine dyes can produce radical ions [38]. Therefore, in order to accurately assess the antioxidant potential of the designed polymers, the dye moiety in the polymer chain was excluded for these experiments.

### Statistical Analyses

The statistical interaction between group means for each time point and treatment group was analyzed by a two-way ANOVA with a Fisher’s Least Significant Difference (LSD) *post hoc* test. There are benefits to using a Fisher’s LSD compared to other multiple comparison tests, including increased power and protection against type I error [39, 40]. Additionally, unlike individual *t*-tests, the Fisher’s LSD test retains power by calculating the pooled standard deviation from all groups rather than only the two groups being analyzed. All analyses were performed using GraphPad Prism software (GraphPad software Inc., La Jolla, CA, USA). A p-value < 0.05 was considered significant.

### Polymer synthesis and characterization

Details of the polymer synthesis and characterization are provided in the Supporting materials file (S1 File).

## Results

### Membrane-reactive polymer synthesis

**Fig 1:**
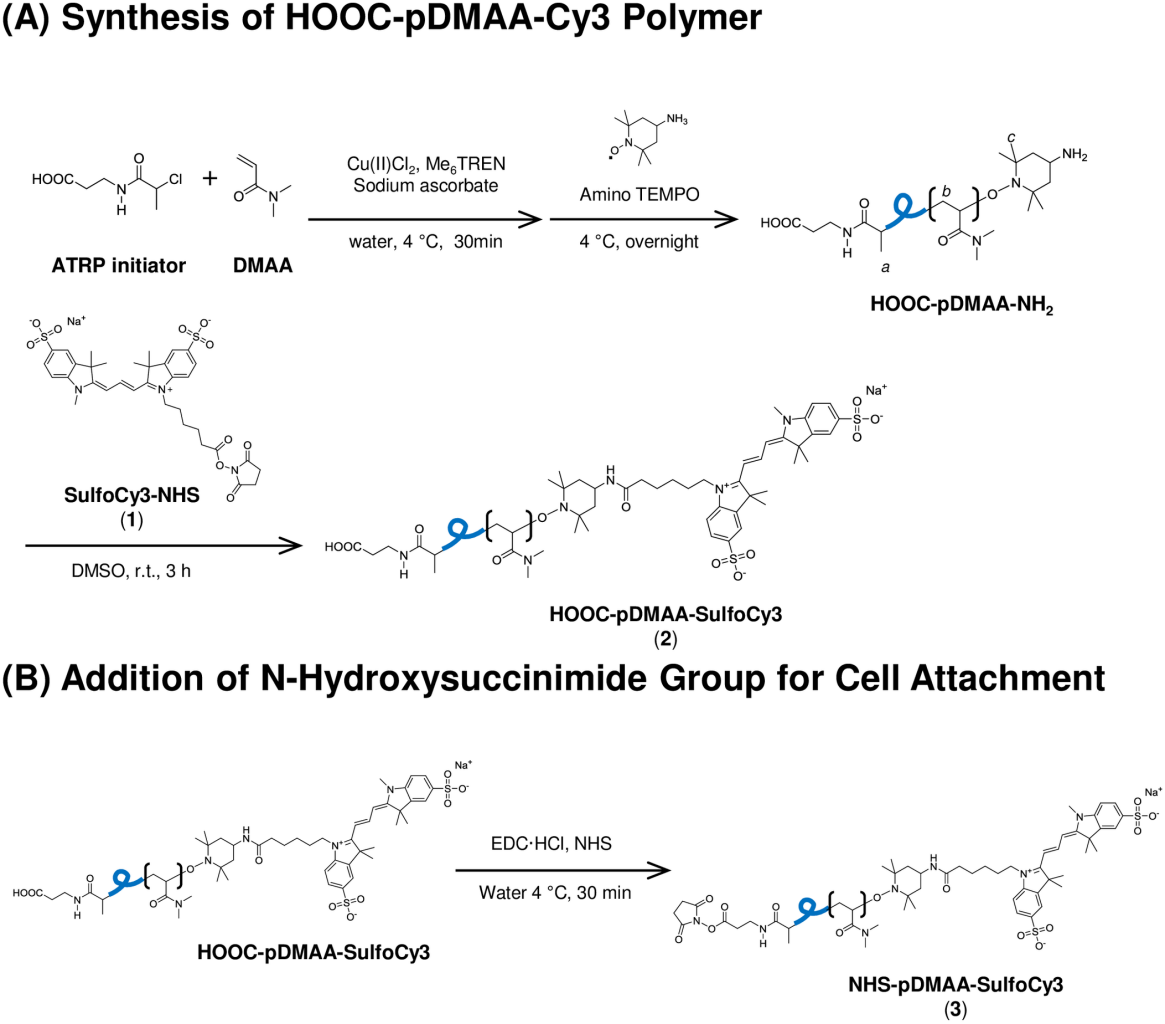
Schematic for the ATRP synthesis of a disulfoCy3-containing poly(DMAA) polymer. Disulfo-Cy3 was added to polymer chains for fluorescence imaging of polymer-exposed cells (**1**). NHS functional group attachment facilitates cell attachment via covalent amide bond formation with surface proteins (**3**). Polymer chains lacking NHS functionality were used as a control for cell surface binding (**2**). The average molecular weight of polymer chains was approximately 7 kDa. Additional information regarding the synthesis of the polymers described in this study can be found in S1 File.

The cell surface binding polymer, NHS-pDMAA-Cy3, (Fig 1) was synthesized by atom-transfer radical polymerization techniques [3] using DMAA as a monomer, *N*-2-chlorolpropionyl-β-alanine as an ATRP initiator, and amino-TEMPO as a termination reagent, followed by reaction with sulfo-cyanine 3 ester and generation of NHS ester on the other polymer end (Fig A in S1 File). A dye moiety (rhodamine or Cy3) was incorporated into the polymer chain in order to use fluorescence to measure the efficiency of membrane engineering and the fate of the polymers that are retained on the cell surface Fig 1.

### The retention of polymers reacted with the membranes of hRBCs

**Fig 2:**
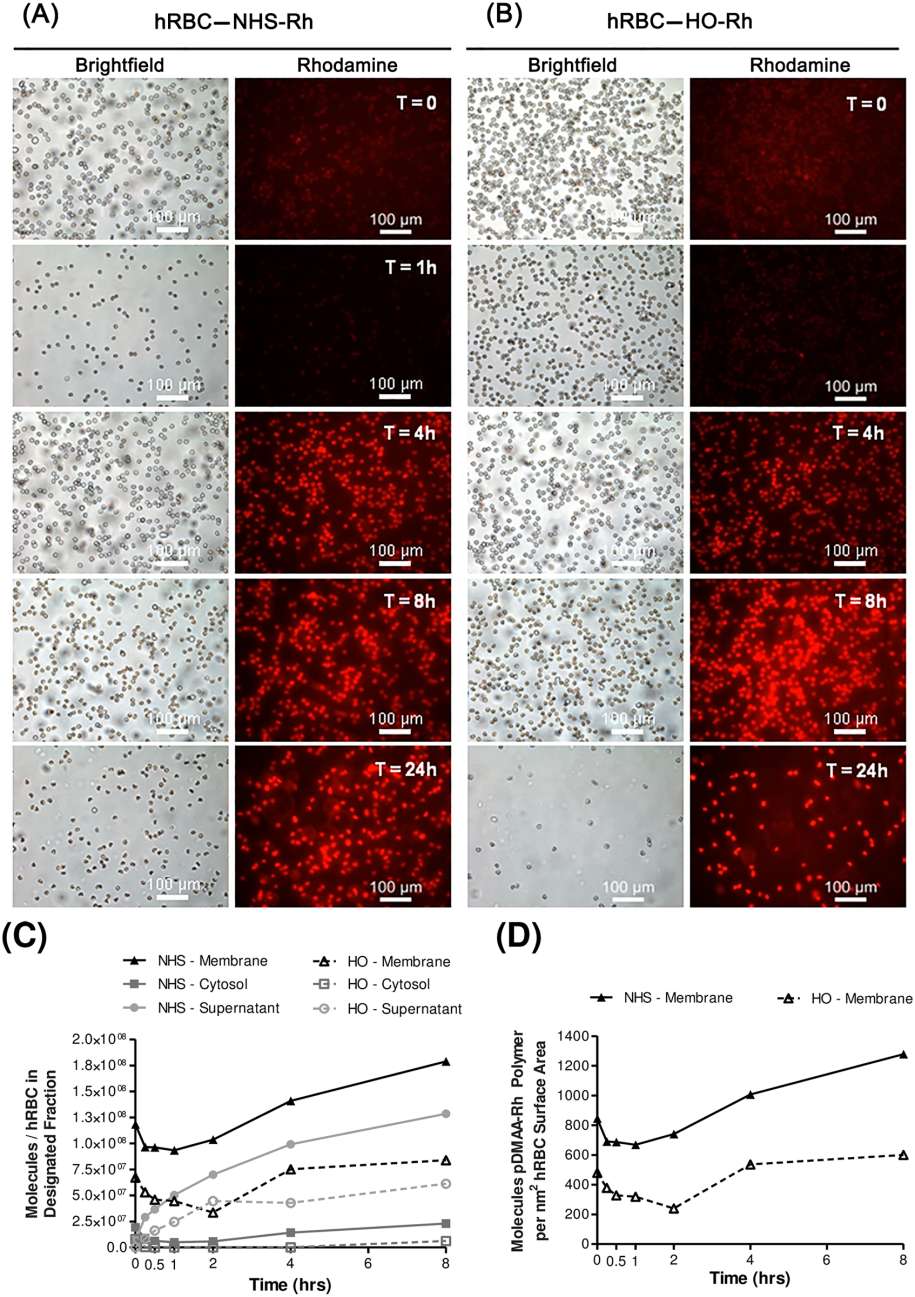
Human erythrocyte membrane engineering with NHS-pDMAA-Rh polymers. NHS-pDMAA-Rh polymers exhibit significant non-specific binding and initial high-density fluorescence quenching. Human RBCs were modified with 182 μM NHS-pDMAA-Rh or 98 μM HO-pDMAA-Rh for 30 minutes at 37°C. (**A**) Representative images of NHS-pDMAA-Rh-exposed hRBC for each designated time point. (**B**) Representative images of HO-pDMAA-Rh-exposed hRBC for each designated time point. Epifluorescent images were capture after washing on a Leica inverted microscope at 20X. Scale bars measure 100 μm. (**C**) Supernatant, cytosolic, and membrane fractions were collected at 0, 1, 4, and 8 hours. Polymer retention and internalization was assessed by monitoring the relative fluorescence of each fraction over time and calculated as the number of polymer molecules per hRBC using a standard curve. (**D**) The number of polymer molecules per nm^2^ hRBC surface area was calculated using an average hRBC surface area of 140 μm^2^. Images were background corrected and the brightness/contrast for each channel was balanced using Image J software. n = 3.

For effective membrane engineering, a delicate balance exists between establishing an optimal surface concentration of polymer chains and reducing untoward effects on the modified cell or significant non-specific binding interactions. In order to determine the retention and fate (i.e., changes in where the polymer resides over time) of polymer chains attached to the erythrocyte surface, microscopy and cell fractionation methods were utilized to measure polymer concentration within the supernatant, the cytosolic compartment, and on the membranes of modified erythrocytes over time. Initial experiments utilized a rhodamine moiety in the polymer chain as a means of fluorescently monitoring membrane engineering. A significant degree of fluorescence self-quenching (0–2 hrs) and rapid fluorescence recovery (2–8 hrs) was observed on the erythrocyte membrane for both polymer molecules (Fig 2C and 2D). Nonetheless, we were disappointed to observe a high degree of non-specific binding with our control (HO-pDMAA-Rh), suggesting a large portion of the polymer retention observed in NHS-pDMAA-Rh-exposed erythrocytes could have been the result of non-specific membrane interactions. Qualitative micrographs confirmed this phenomenon, and by 24 hours ghost formation was observed in both polymer groups (Fig 2A and 2B). We report the data here since many investigators are using rhodamine-labeled molecules to track apparently specific binding events. The non-specific binding of rhodamine to cells and proteins is likely deserving of further study. We next designed an alternative reactive polymer with greater hydrophilicity in the fluorescent tag. The disulfo-Cy3 moiety was more soluble in buffer solutions than the underivatized Cy3 and the non-reactive control polymer exhibited less non-specific interaction with the cell surface than the more hydrophobic rhodamine. Dye hydrophobicity has been correlated with the potential for non-specific binding, although as mentioned above, many published studies use rhodamine under the assumption that it does not associate with the cell membrane [3].

**Fig 3:**
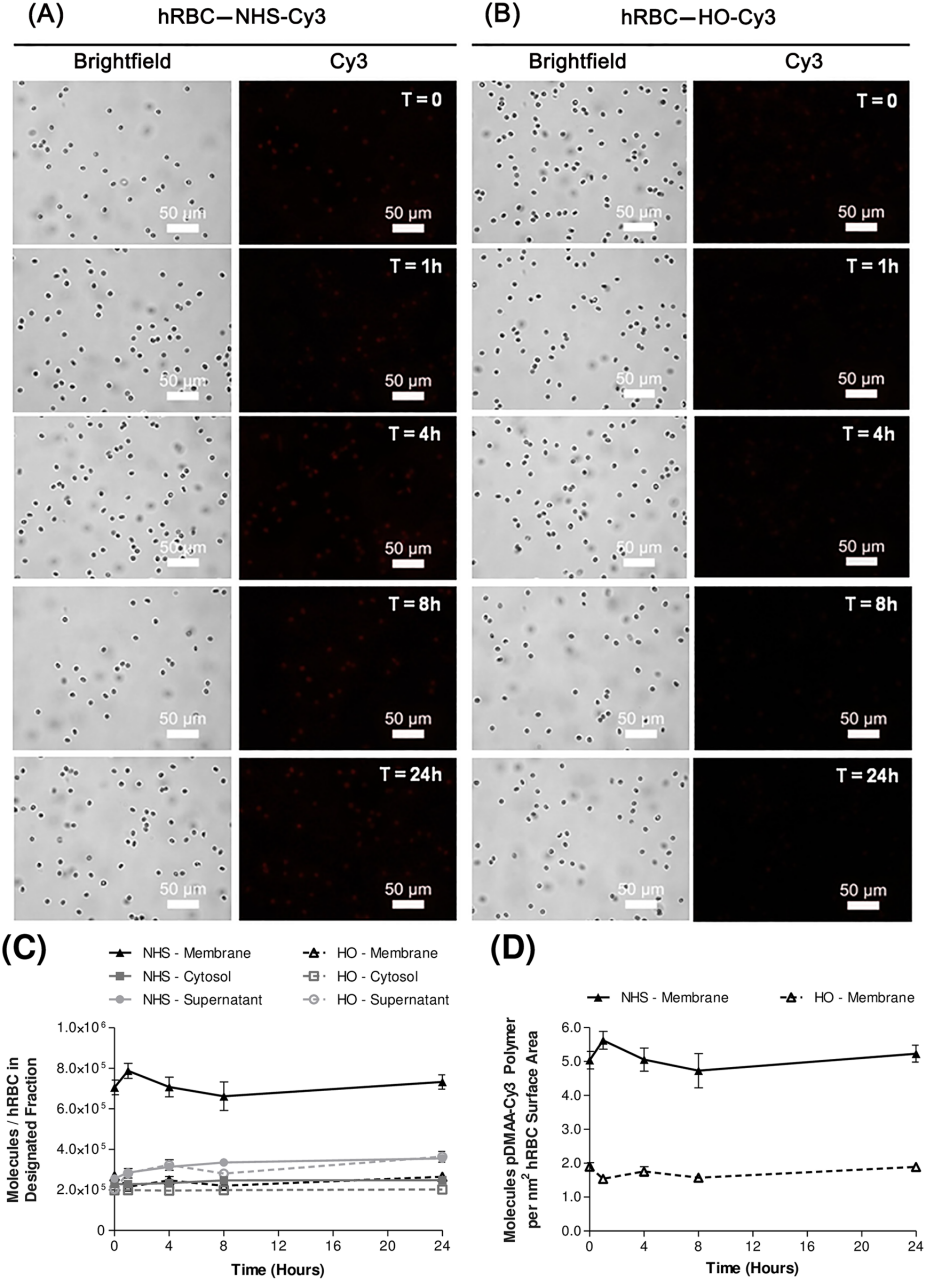
Human erythrocyte membrane engineering with NHS-pDMAA-Cy3 polymers. NHS-pDMAA-Cy3 polymers are retained on hRBC membranes over a 24 hour period compared to HO-pDMAA-Cy3 polymers, indicating cell surface reactivity. Human RBCs were modified with 100 μM NHS-pDMAA-Cy3 or HO-pDMAA-Cy3 for 30 minutes at 37°C. (**A**) Representative images of NHS-pDMAA-Cy3-exposed hRBC for each designated time point. (**B**) Representative images of HO-pDMAA-Cy3-exposed hRBC for each designated time point. Epifluorescent images were capture after washing on a Leica inverted microscope at 40X. Scale bars measure 50 μm. (**C**) Supernatant, cytosolic, and membrane fractions were collected at 0, 1, 4, 8, and 24 hours. Polymer retention and internalization was assessed by monitoring the relative fluorescence of each fraction over time and calculated as the number of polymer molecules per hRBC using a standard curve. (**D**) The number of polymer molecules per nm^2^ hRBC surface area was calculated using an average hRBC surface area of 140 μm^2^. Images were background corrected and the brightness/contrast for each channel was balanced using Image J software. n = 3.

Reactive pDMAA-Cy3 polymers containing a pendant NHS group were retained significantly longer on erythrocytes than the non-reactive control polymer (Fig 3A). Most of the residual control polymer was quickly washed away in the supernatant of initial rinses (Fig 3B). Quantification of Cy3 fluorescence in collected fractions was used to measure the efficiency of reactive membrane engineering and the fate of the attached polymers over time. Using fluorescence analyses, we showed that hRBCs exposed to NHS-pDMAA-Cy3 resulted in an initial covalent binding of 7.1 *×* 10^5^ molecules/cell on the membrane (Fig 3C). The initial concentration of polymers in the supernatant fractions were similar between HO-pDMAA-Cy3- and NHS-pDMAA-Cy3-exposed hRBCs (2.0 *×* 10^5^ and 2.6 *×* 10^5^ molecules/cell, respectively); however, there was a nearly 3-fold decrease in HO-pDMAA-Cy3 polymer concentration on the membrane compared to the membrane concentration in NHS-pDMAA-Cy3-exposed cells (2.7 *×* 10^5^ molecules/cell versus 7.1 *×* 10^5^ molecules/cell, respectively) (Fig 3C). Moreover, the signal for NHS-pDMAA-Cy3-modified hRBCs remained relatively consistent over a 24 hour period, with a final membrane fraction concentration of 7.3 *×* 10^5^ molecules/cell. Human RBCs exposed to HO-pDMAA-Cy3 demonstrated a greater loss of polymer in the membrane fraction over time (-1.6 *×* 10^5^ molecules/cell). Accounting for non-specific binding in the control polymer group and an average erythrocyte size of 140 μm^2^ [35], the density of NHS-pDMAA-Cy3 molecules on hRBC membranes was approximated at 3 polymer molecules per nm^2^ (Fig 3D). Translocation of polymer to the cytosolic compartment was minimal; 1.4 *×* 10^5^ molecules for NHS-pDMAA-Cy3-modified hRBCs after correcting for non-specific interactions in the control group. Lastly, the potential to modify erythrocyte ghosts with NHS-pDMAA-Cy3 was examined.

**Fig 4:**
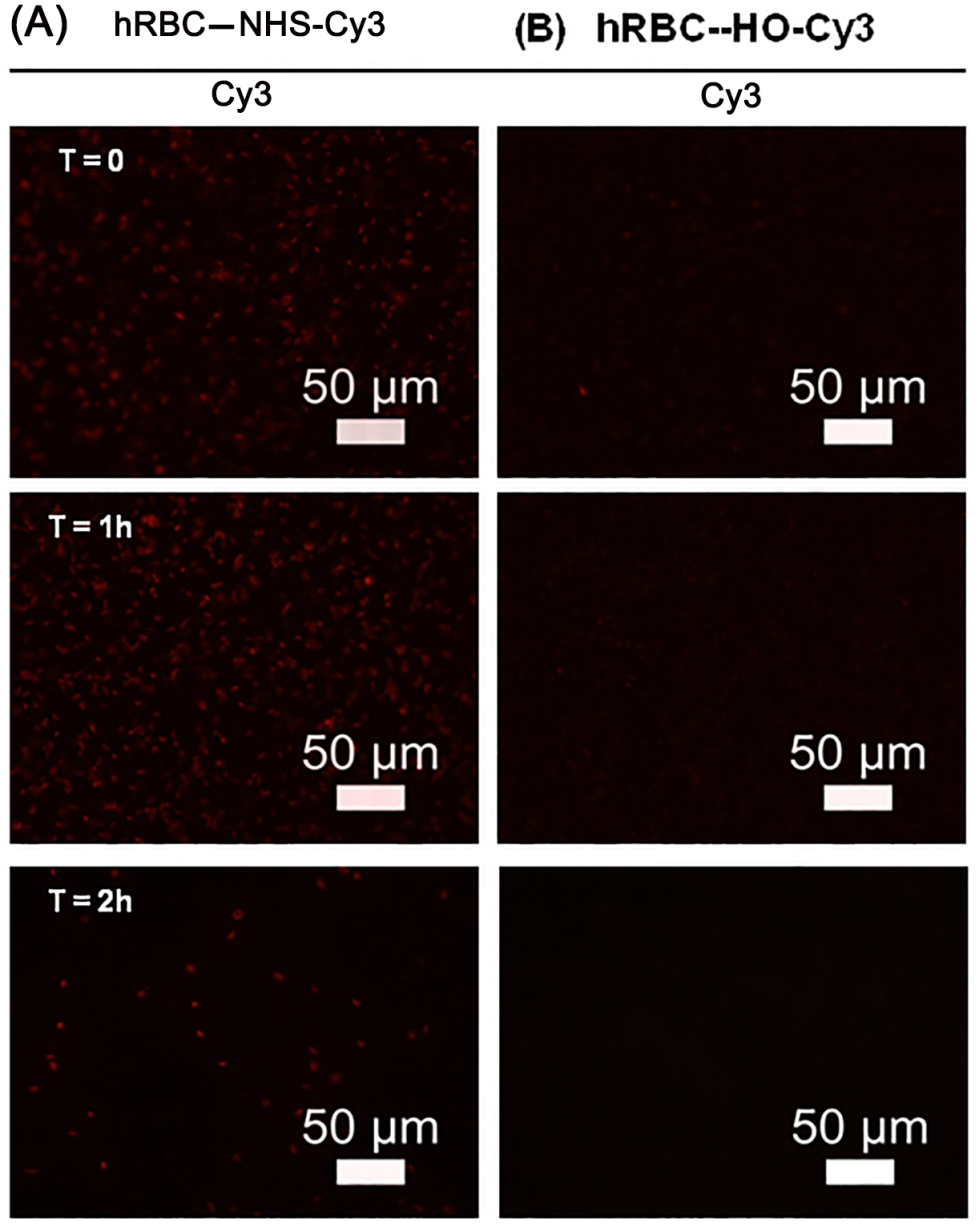
Human ghost erythrocyte membrane engineering with NHS-pDMAA-Cy3 polymers. Human erythrocyte ghosts can be surface modified with NHS-pDMAA-Cy3 and exhibit short-term retention of polymer. Ghost membranes were produced from intact hRBCs using hypotonic methods. Human RBC ghosts were exposed to either 100 μM NHS- or HO-pDMAA-Cy3 polymers for 30 minutes at 37°C. Epifluorescent images were captured after washing using an inverted Leica microscope at 40X. hRBC exposed to control polymer demonstrate negligible fluorescent signal (**B**) while those exposed to NHS-pDMAA-Cy3 exhibit strong fluorescence that diminishes over 2 hours (**A**). Representative images are shown for each time point. Scale bars measure 50 μm. Images were background corrected and the brightness/contrast for each channel was balanced using Image J software.

Although erythrocyte ghost were adequately modified using the procedure for intact red blood cells, the lifetime of polymer chains on the surface was significantly shortened to two hours (Fig 4). Overall, these data support the utility of ATRP methods as a means of creating cell surface reactive polymers with adequate and consistent surface retention and minimal residual non-specific binding to human red blood cells.

### The impact of membrane engineering on Band 3 protein aggregation under physiological conditions

**Fig 5:**
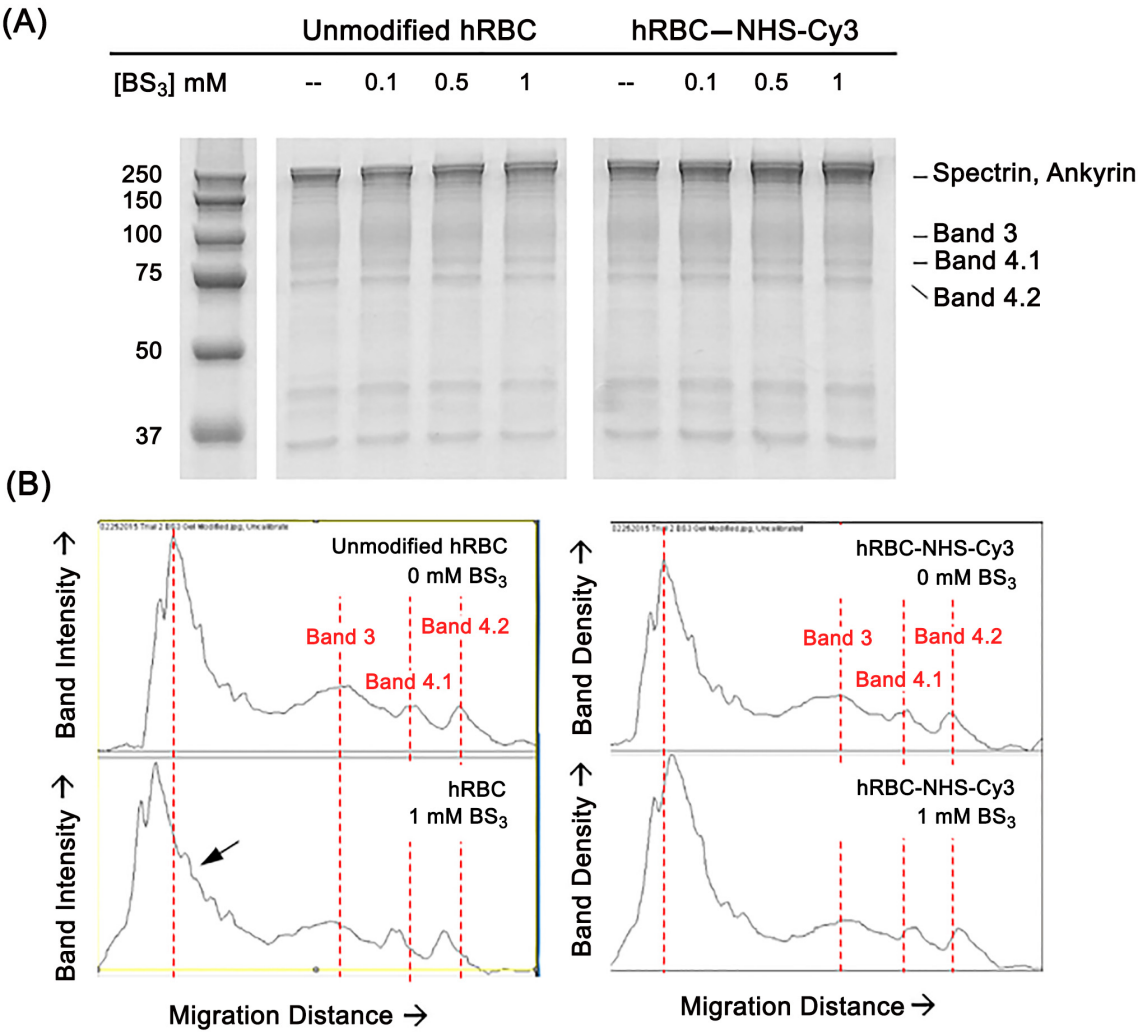
The effect of human erythrocyte membrane engineering with NHS-pDMAA-Cy3 polymers on Band 3 aggregation. Human erythrocyte Band 3 protein is a major site of NHS-pDMAA-Cy3 modification supported by the absence of Band 3 aggregate formation in the presence of the protein cross-linker bis(sulfosuccinimidyl)suberate (BS_3_). (**A**) Isolated hRBC membranes for each group were run on 12% TGX polyacrylamide gels and stained with Imperial protein stain. Gel image truncated after 37 kDa molecular weight marker to highlight larger molecular weight membrane proteins. (**B**) Histogram plots of band intensity versus migration distance were plotted for each experimental group and concentration (0 mM and 1 mM BS_3_ concentrations shown). Dotted lines are used to represent peak intensity of specific protein bands in non-crosslinked hRBC membranes. Specifically, at the highest concentration of BS_3_ (1 mM) there is a noticeable decrease in monomeric Band 3 protein band intensity as well as a modest increase in higher molecular weight proteins gathering at the top of the gel (e.g., in the region of spectrin and ankyrin proteins) for unmodified hRBCs versus those modified with NHS-pDMAA-Cy3 polymers. The arrow represents Band 3 aggregates.

The integral membrane protein Band 3 comprises approximately 25% by weight of the protein content in erythrocytes with nearly 1 million copies within the membrane [28, 41, 42]. As such, it has been used as a site of biotinylation, pegylation, and crosslinking [20, 42, 43]. Band 3 lateral mobility and connection to intracellular linking proteins such as ankyrin and Band 4.1 is essential for controlling the morphology and clearance of red blood cells in circulation [4, 44, 45]. Band 3 clustering and aggregation, either by oxidation or cross-linking is implicated in clearance mechanisms [4–6, 46]. Therefore, it was essential to determine the impact that covalent attachment of ATRP-synthesized polymers had on this erythrocyte protein; more specifically, whether membrane engineering modified Band 3 protein aggregation under physiological conditions. Unmodified or polymer-modified hRBCs were treated with varying concentrations of the membrane impermeable crosslinking agent bis(sulfosuccinimidyl)suberate (BS_3_). Immediately following modification, treatment, and washing, hRBC ghosts were produced as described and subjected to SDS-PAGE following the methods of Laemmli [36]. Similar to studies examining the effect of crosslinker-induced aggregation of Band 3 [43, 47], the pattern of membrane protein banding was compared between unmodified and modified hRBC by examining mobility shifts on the resulting gels. Specific attention was focused on Band 3 migration around its apparent molecular weight of approximately 100 kDa [48] and the formation of Band 3 aggregates (e.g., dimers, trimers, etc.). Representative SDS-PAGE results (Fig 5A) were analyzed quantitatively with Image J and graphed as shown in Fig 5B. Noticeable electrophoretic mobility shifts were discovered in unmodified hRBCs, particularly at the highest BS_3_ concentration of 1 mM. Using the red dotted line in Fig 5B as a reference point for untreated cells, distinctive shifts in the apparent molecular weight of Band 3 protein and those in the area of spectrin and ankyrin were prominent. Moreover, while dose-dependent alterations to monomeric Band 3 and aggregate formation were noticeable in unmodified hRBCs, similar changes were absent in the banding patterns of hRBC modified with NHS-pDMAA-Cy3 polymer chains. these data suggest that primary amines in the human erythrocyte Band 3 protein may have been major sites of NHS-pDMAA-Cy3 modification and that modification at these sites may have the potential to mitigate the formation of Band 3 aggregates in the presence of the protein crosslinker BS_3_.

### Effects of membrane engineering on hRBC membrane stability under physiological and oxidizing conditions

Under normal physiological conditions, phosphatidylserine (PS) is relegated to the inner leaflet of the erythrocyte membrane. Both oxidative damage to the erythrocyte membrane and alterations to proteins responsible for lipid translocation have been implicated in PS exposure in the outer membrane leaflet [17]. Therefore, the impact of surface polymer modification on the membrane stabilization of modified human erythrocytes was examined using a commercial kit designed to track PS externalization by fluorescence microscopy and flow cytometry.

**Fig 6:**
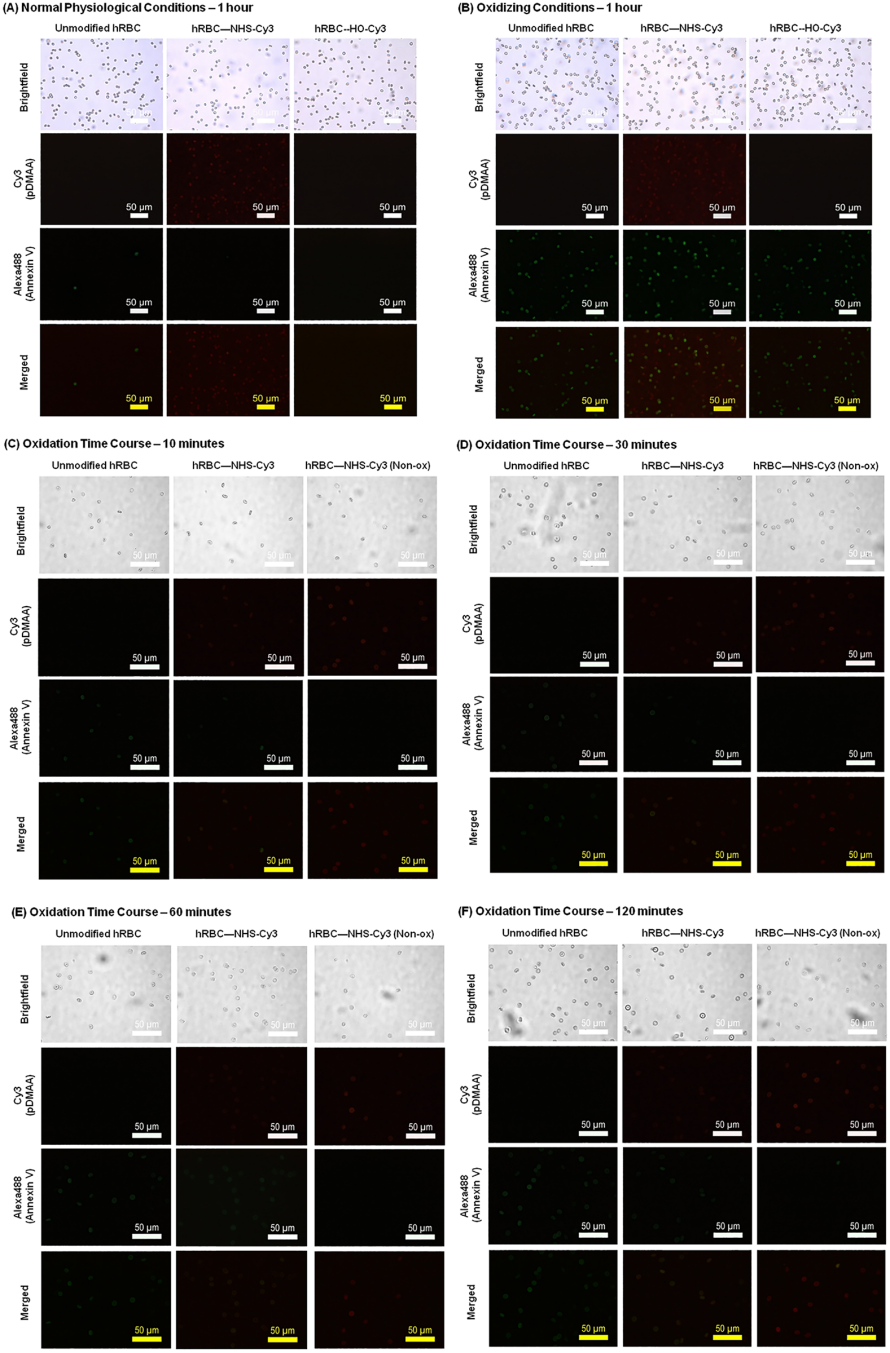
Effect of human erythrocyte membrane engineering with NHS-pDMAA-Cy3 polymers on membrane stability. (**A**) Modification of hRBC with NHS-pDMAA-Cy3 polymers does not induce membrane destabilization as evidenced by the absence of PS-Annexin V signal under physiological conditions. Both unmodified and modified (100 μM polymer solution) hRBC were incubated in 1X PBS for one hour at 37°C. Annexin V-Alexa488 binding to externalized phosphatidylserine was used to monitor membrane destabilization and oxidative damage (green fluorescence). Cy3-modification demonstrated by red fluorescence. Epifluorescent images of PS-Annexin V and Cy3 channels captured under oil at 60x using an inverted Leica microscope. Individual channels were merged using Image J. Scale bars measure 50 μm. Images were background corrected and the brightness/contrast for each channel was balanced using Image J software. (**B**) Effect of human erythrocyte membrane engineering with NHS-pDMAA-Cy3 polymers on membrane damage during oxidant exposure. Modification of hRBC with NHS-pDMAA-Cy3 polymers may protect against membrane damage under oxidizing conditions demonstrated by a potential mitigation of phosphatidylserine-Annexin V signal. Both unmodified and modified (100 μM polymer solution) hRBC were incubated in 0.2 mM CuSO_4_/2.5 mM ascorbate solution for one hour at 37°C. Annexin V-Alexa488 binding to externalized phosphatidylserine was used to monitor membrane destabilization and oxidative damage (green fluorescence). Cy3-modification demonstrated by red fluorescence. Epifluorescent images of PS-Annexin V and Cy3 channels captured under oil at 60X using an inverted Leica microscope. Individual channels were merged using Image J. Scale bars measure 50 μm. Images were background corrected and the brightness/contrast for each channel was balanced using Image J software. (**C-F**). Time point analysis of the effect of human erythrocyte membrane engineering with NHS-pDMAA-Cy3 polymers on membrane damage during oxidant exposure. Modification of hRBC with NHS-pDMAA-Cy3 polymers may protect against membrane damage under oxidizing conditions demonstrated by a potential mitigation of phosphatidylserine-Annexin V signal over time. To further investigate the temporal regulation of these events, both unmodified and modified (100 μM polymer solution) hRBC were incubated in 0.2 mM CuSO_4_/2.5 mM ascorbate solution for 10 (**C**), 30 (**D**), 60 (**E**), or 120 (**F**) minutes at 37°C. Annexin V-Alexa488 binding to externalized phosphatidylserine was used to monitor membrane destabilization and oxidative damage (green fluorescence). Cy3-modification demonstrated by red fluorescence. Epifluorescent images of PS-Annexin V and Cy3 channels captured under oil at 60X using an inverted Leica microscope. Individual channels were merged using Image J. Scale bars measure 50 μm. Images were background corrected and the brightness/contrast for each channel was balanced using Image J software.

Preliminary experiments showed that exposure of human erythrocytes to Cy3-containing polymers under normal physiological conditions (i.e., isotonic PBS, pH 7.4) did not result in significant PS exposure after incubation up to one hour (Fig 6A), suggesting that the polymers did not inflict membrane damage that would result in PS exposure and clearance. When incubated in oxidizing conditions (induced with 0.2 mM CuSO_4_/2.5 mM ascorbate) for an identical time, hRBCs exposed to control polymer (HO-pDMAA-Cy3) demonstrated PS exposure identified by Alexa488-Annexin V fluorescence similar to unmodified, oxidized hRBCs (Fig 6B). Interestingly, membrane modification of hRBC with NHS-pDMAA-Cy3 polymers also impacted the degree of oxidation evidenced by an apparent reduction in Alexa488-Annexin V fluorescence (Fig 6C–6F). It should not be surprising that the introduction of a large number of oxidizable polymers on the surface of a cell would in and of itself absorb some degree of oxidative power. Unfortunately, we are unable to isolate and characterize the polymers once they have reacted with the cell membrane to confirm this mechanism of stabilization. Although surface modification of hRBCs induced moderate changes in morphology (i.e., echinocyte formation), the methods were otherwise biocompatible. Thus, it appeared that NHS-pDMAA-Cy3 polymers had the potential to protect hRBCs in a pro-oxidant environment. Flow cytometry was used to further explore this phenomenon as explained in Section 3.5.

### Effects of membrane engineering with antioxidant (TEMPO)-containing polymers on hRBC membrane stability under physiological and oxidizing conditions

We exposed modified and normal hRBCs to hydroxyl radicals formed by the *in situ* reaction of ascorbate and copper II [10]. These highly reactive radicals can induce oxidative damage to lipids, proteins, and nucleic acids [10, 11, 49]. Although we observed PS externalization for both unmodified erythrocytes and those modified with NHS-pDMAA-Cy3 in the oxidizing environment (Fig 6B–6F), modification with NHS-pDMAA-Cy3 appeared to mitigate PS externalization over time, suggesting a potentially time-dependent cytoprotective effect. As such, we were interested in exploring whether the inclusion of antioxidant polymers might enhance this effect.

**Fig 7:**
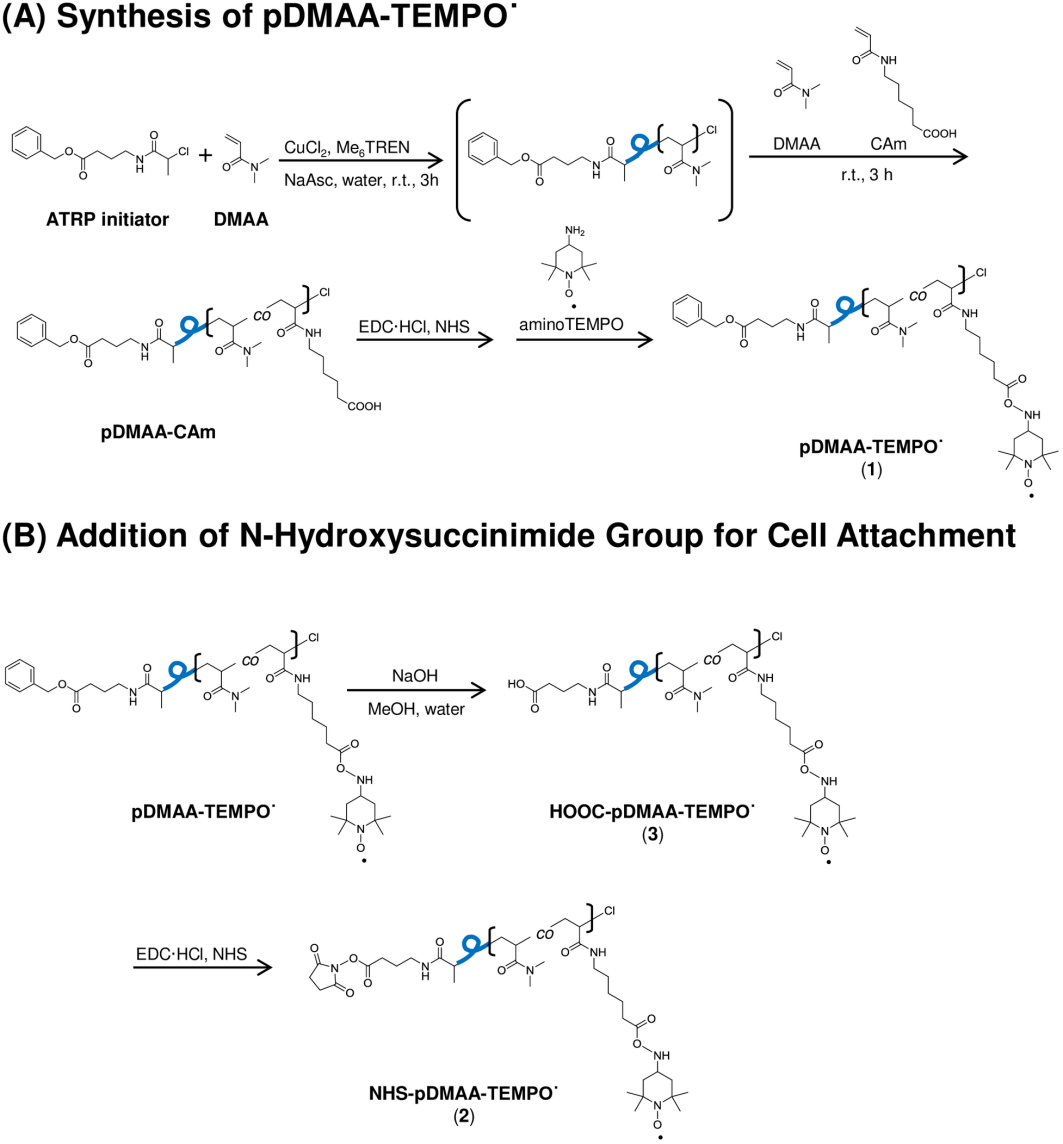
Schematic for the ATRP synthesis of a TEMPO-terminated poly(DMAA) polymer. The stable free-radical nitroxide, 4-amino-2,2,6,6-tetramethylpiperidine-1-oxyl (TEMPO˙) was added to polymer chains to investigate its effects as a cytoprotective antioxidant attached to human red blood cells (**1**). NHS functional group attachment facilitates cell attachment via covalent amide bond formation with surface proteins (**2**). Polymer chains lacking NHS functionality were used as a control for cell surface binding (**3**). The average molecular weight of polymer chains was approximately 6.2kDa.

**Fig 8:**
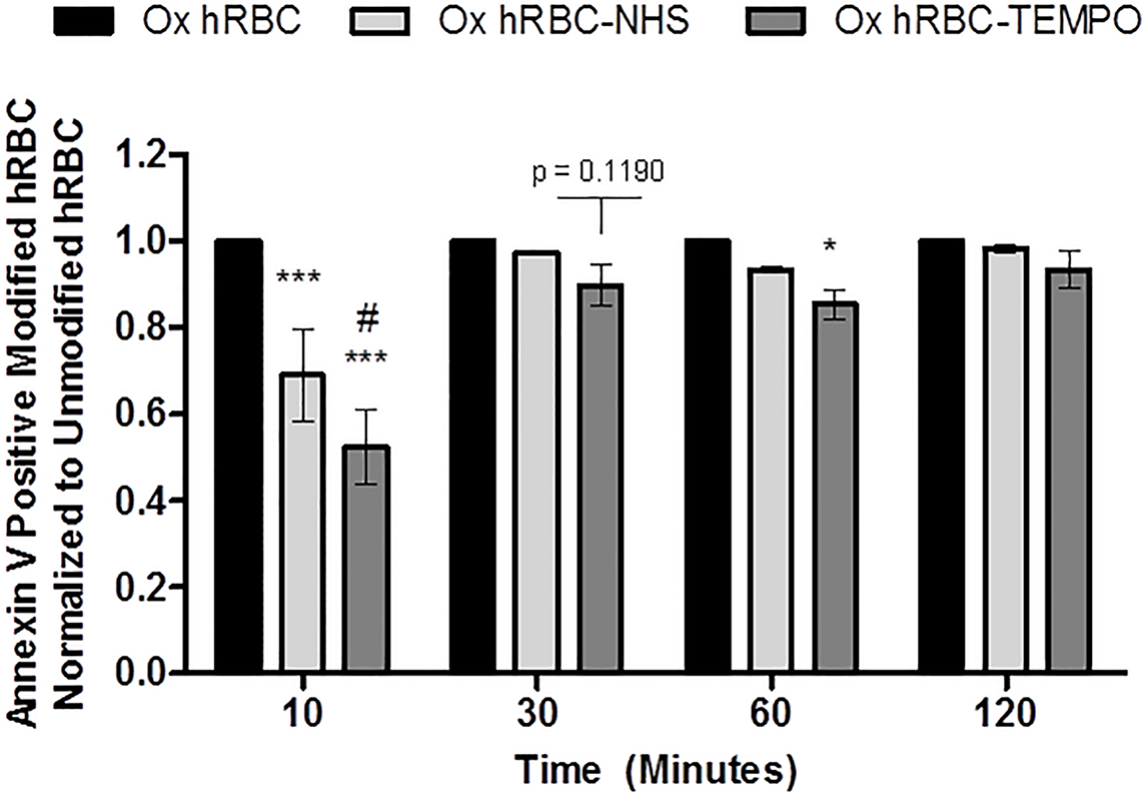
Effect of human erythrocyte membrane engineering with NHS-pDMAA-TEMPO˙ polymers on membrane damage during oxidant exposure over time. Human erythrocytes modified with either NHS-pDMAA-Cy3 or NHS-pDMAA-TEMPO˙ demonstrated significant or nearly significant cytoprotective trends over time when exposed to oxidizing conditions (0.1 mM CuSO_4_/1.25 mM ascorbate solution). Results are normalized to unmodified, oxidized hRBCs for each time point. Oxidation in this assay is indirectly measured by monitoring externalized phosphatidylserine tagging by Annexin V-Alexa488 using flow cytometry. Cell populations were gated as either positive or negative for Annexin V-Alexa488 fluorescence. *** indicates significance, p < 0.001 compared to unmodified hRBCs; * indicates significance, p < 0.05 compared to unmodified hRBCs; # indicates significance, p < 0.05 compared to NHS-pDMAA-Cy3 modified hRBCs. NHS-pDMAA-TEMPO˙-modified hRBCs are nearly significantly different to unmodified hRBCs at 30 minutes (p = 0.1190). Results were analyzed by a two-way ANOVA with Fisher’s LSD *post hoc* test. Bars represent group means ± SEM. n = 3.

The cell-permeable, stable nitroxide radical TEMPO˙ (2,2,6,6-tetramethylpiperidin-1-yl)oxyl) and its hydroxylated form, Tempol (4-hydroxy-TEMPO), have illustrated significant antioxidant effects, including the neutralization of hydroxyl radicals [24–27]. Thus, given the potential radical scavenging activity of TEMPO in our model system, hRBCs modified with NHS-pDMAA-TEMPO˙ polymer chains were generated to determine the degree of PS exposure in these oxidant-challenged erythrocytes (Fig 7). Details of the synthetic procedure and characteristics of NHS-pDMAA-TEMPO˙ are described in Supporting Materials (Fig E in S1 File).^1^H NMR analysis determined that the polymer contained 3.1 of TEMPO units per polymer chain (i.e. 6.8% w/w TEMPO in the polymers). Our results from flow cytometry supported the qualitative trends in reduced PS externalization noted from microscopy for NHS-pDMAA-Cy3-modified hRBCs. Further, NHS-pDMAA-TEMPO˙ modification of hRBCs also mitigated PS externalization suggesting the potential to protect hRBCs in an pro-oxidant environment by indirectly monitoring PS externalization (Fig 8). The greatest significance for both polymer types was seen at the earliest time point (10 minutes), but was quickly lost over time for NHS-pDMAA-Cy3-modified hRBCs. Conversely, a significant mitigation of oxidative damaged continued for NHS-pDMAA-TEMPO-modified hRBCs for up to 1 hour under oxidizing conditions. The extended effects of NHS-pDMAA-TEMPO-modified hRBCs were likely due to the greater neutralization of hydroxyl radicals and reduction in PS externalization at 10 minutes versus those of the NHS-pDMAA-Cy3-modified group.

## Discussion

Despite their vital function, mature erythrocytes have a limited capacity to repair cellular damage and are unable to synthesize new proteins to replace those that are irreversibly damaged by oxidative stress [45, 50]. Thus, the average lifespan of a human red blood cell in circulation is limited to 120 days. There is evidence that erythrocytes undergo oxidative changes in conditions where free radicals are in high concentrations such as rheumatoid arthritis, diabetes, inflammation, and cancer [8, 9, 45]. In healthy erythrocytes, enzymatic and non-enzymatic components efficiently neutralize oxidative damage; however, their supply is finite and consumable. Erythrocytes from patients with sepsis, sickle cell disease, thalassemia, glucose-6-phosphate dehydrogenase deficiency, and phosphate depletion have an increased sensitivity to apoptotic stimuli, and subsequently, a shortened lifespan [45]. Moreover, membrane lipid disorders can lead to premature removal as well as vascular dysfunction [45]. Increased ROS production in end-stage renal patients was associated with membrane cytoskeleton instability, loss of erythrocyte surface area, and transformation of normal discocytes [45]. These studies suggest that the preservation or substitution of cytoprotective mechanisms may be of benefit and maintain adequate circulation half-life. As a result of increased reactive oxygen species (ROS) production or the exhaustion of endogenous antioxidant mechanisms, the circulating erythrocyte becomes a primary target of oxidative damage. As such, the integrity of the erythrocyte membrane becomes a critical lynchpin to its survival in circulation. Thus, our goal was to study the fate of polymers covalently attached to human erythrocytes and to determine their effect on cell stability under physiological and oxidative conditions *in vitro*.

### Surface Retention and Translocation of Cy3-containing Polymers in Human Erythrocytes

Fresh, packed, leukocyte-reduced, Rh-positive human erythrocytes from healthy volunteers were modified via NHS-terminated polymer binding to surface proteins (Figs 1 and 7). The average molecular weight of linear polymers synthesized in this study was 7 kDa, similar to the most commonly used linear PEG polymers of 5 kDa [51]. Azim-Zadeh and colleagues used biotin derivatives, such as sulfo-NHS-LC-biotin, to probe conformation changes in Band 3 proteins in normal and malaria parasite-infected erythrocytes. Although there are 29 total lysine residues within the transmembrane domains of Band 3, only 9 lysines are exposed within two extracellular loops [42]. The enhanced conformational flexibility of the major flexible extracellular loop and the high proportion of hydrophobic regions may explain the finding that only 2 lysines in the extracellular domain had been biotinylated in normal erythrocytes [42]. Biotinylation of mouse erythrocytes evaluated by quantifying bound streptavidin accounted for only 6 *×* 10^5^ molecules/erythrocyte [43] and the binding density of avidin to biotin-phosphatidylethanolamine was approximated at 5 *×* 10^5^ molecules/red blood cell [52]. Indeed, the degree of modification in our study was identical to the latter (approximately 5 *×* 10^5^ NHS-pDMAA-Cy3/cell). Nonetheless the degree of surface polymer modification in erythrocytes has been shown to be species-dependent [47], correlated with polymer structure, and to directly influence RBC clearance [49]. This is evidenced by the effect of different dye moieties on the degree of non-specific binding observed in our study (Fig 2). The hydrophobic nature of rhodamine contributed to a greater degree of non-specific binding that was not removed during washing procedures. The increase in fluorescent intensity over time may be attributed to collisional and aggregate self-quenching of rhodamine at high surface concentrations immediately following membrane modification [41]. As some of the rhodamine-containing polymer was slowly released from the erythrocyte surface over time, self-quenching was relieved, but non-specific interactions between the released polymer dye and the membrane may have continued. This may have caused the unexplained increase in fluorescent intensity that was observed (Fig 2A–2C). Moreover, this data may provide important insight to researchers using rhodamine-labeled molecules to track specific binding events. Conversely, the hydrophobic interaction between Cy3 and the erythrocyte membrane was likely subdued by the sulfo groups, which confer some hydrophilicity to the dye. Although non-specific binding in the Cy3 control polymer-exposed hRBCs was relatively low, the observed binding in NHS-pDMAA-Cy3-modified hRBCs was likely still a mixture of specific and non-specific interactions, however small. This suggests that other chemical-biological interactions such as polymer chain length or steric hindrance may influence membrane engineering. While the polymers utilized in this study were relatively short in length, our previous report of surface medication of mesenchymal stem cells suggests that polymer length may affect the efficiency of targeting various tissues [30]. Thus, extending the polymer chain length in future studies could be beneficial for the interaction of hRBCs carrying targeting ligands to their tissue target or target cells; something the authors are already considering for future experiments.

Modification of hRBCs with 100 μM NHS-pDMAA-Cy3 polymer for a short period of time (i.e., 30 minutes) resulted in robust, homogenous fluorescence at the periphery of the cells and a relatively consistent retention on the membrane for at least 24 hours (Fig 3C). In fact, polymer density in membrane fractions appeared to stabilize by 4 hours. The covalent interaction between NHS and primary amines in membrane proteins likely contributed to the quick, stable reaction of polymers with the erythrocyte surface. Albeit time point analysis was extended to 4 days, Cy3 fluorescence was markedly reduced and ghost formation was evident, suggesting amide bond hydrolysis by proteases, either secreted or within the lysosomal compartment. Additionally, low but detectable fluorescence was measured in the cytosolic fractions from modified hRBCs. These observations are similar to the modification of human embryonic kidney (HEK293) and human leukemic lymphoblast (CCRF-CEM) cell lines with 5 kDa NHS-FITC-PEG polymers [12]. Despite covalent modification, retention of NHS-FITC-PEG polymers on the cell surfaces was transient and almost completely lost by 48 hours. Moreover, internalization of NHS-FITC-PEG was minimal, but apparent in both cell lines. Some NHS-terminated polymers are membrane permeable [13] and endocytosis of polymer chains or polymer chain components via hydrophobic interactions cannot be completely ruled out. Thus, our observations using a similarly sized, fluorescently tagged, NHS-terminated polymer are supported by these reports.

Moderate echinocyte formation was noted in microscopic examination of NHS-pDMAA-Cy3-modified cells, although this is not uncommon with this method of surface modification. The bilayer-couple hypothesis of erythrocyte morphology suggests that any effect that expands the outer leaflet of the erythrocyte surface relative to the inner leaflet produces a tendency to form echinocytic spicules to accommodate the extra area [39, 40]. Fisher and colleagues noted that the 5 kDa active linear PEG modification of erythrocytes at concentrations of 10 mM resulted is some abnormalities in cell morphology [53]. Similar studies identified a concentration-dependent effect of mPEG polymer coating concentration on echinocyte formation in modified erythrocytes [42, 51]. It is important to mention that significantly deformed cells would have reduced deformability and had a greater potential for getting trapped in the microcirculation [54]. Additionally, large surface concentrations of PEG are associated with erythrocyte aggregation [21, 55]. The initial coating concentration utilized in this study was significantly lower than those mentioned above and erythrocyte aggregation was not observed following surface modification. As such, echinocytosis in hRBC modified with NHS-pDMAA-Cy3, although apparent, may have less severe consequences on erythrocyte functionality in circulation than other modification methods.

### Effect of Polymer Membrane Engineering on Band 3 Protein in Human Erythrocytes

Since changes to erythrocyte morphology are intimately linked to membrane proteins; cytoskeletal arrangement; and membrane phospholipid dynamics; the effect of polymer modification on the major integral erythrocyte protein, Band 3, and a prominent marker of membrane destabilization, phosphatidylserine (PS) externalization, were examined. Band 3 is a well-known site of biotinylation, pegylation, and crosslinking [20, 42, 43]; and conformational changes in Band 3 protein influences the shape of human erythrocytes [43]. For example, the application of crosslinking agents specific to Band 3 induced an echinocytic shape that was reversed with application of competitive inhibitors [43] and treatment of human erythrocytes with 3 mM BS_3_ was shown to enhance the quantity of Band 3 plus spectrin aggregates in the membrane [20]. Similarly, in murine erythrocytes, the degree of Band 3 protein crosslinking induced by 5 mM BS_3_ was approximated at 28–29% [43]. Although a lower concentration of BS_3_ was used than reported in these studies, analysis of membrane proteins in unmodified hRBCs treated with BS_3_ may demonstrate a concentration-dependent increase in Band 3 dimerization, supported by the merging of Band 3 dimers with higher molecular weight proteins such as ankyrin and spectrin near the top of the gel (Fig 5A and 5B). Additionally, Marczak and Józwiak discovered that low concentrations of the crosslinking agent gluteraldehyde induced significant protein aggregation in the region of spectrin with concomitant reductions in monomeric Band 3 [47]. In contrast to unmodified hRBCs, modification of hRBCs with NHS-pDMAA-Cy3 prior to BS_3_ exposure appeared to mitigated the formation of crosslinked aggregates (Fig 5A and 5B). In order to determine polymer interaction with individual membrane-associated proteins in addition to Band 3, such as Band 4.1, Band 4.2, and glycophorin A, the samples were run under reducing conditions. This may have disrupted the disulfide linkages purported to be important in Band 3 aggregate formation, making the banding for dimers and trimers less apparent that if run under native conditions. While analyzing polymer-Band 3 interactions under non-reducing conditions may have better identified the presence of Band 3 aggregates and their mitigation by NHS-polymer hRBC surface modification, it may not have revealed the potential interaction of NHS-polymers with other individual membrane-associated proteins (e.g., Protein 4.1, Protein 4.2, glycophorin A, etc.), which was of primary interested to the authors for this study. Under native condition, these individual proteins would have likely formed larger protein complexes, making it more difficult to determine the protein interactions involved in NHS-polymer membrane binding. It may appear that the shifts in mobility between unmodified and polymer-modified hRBCs with increasing concentrations of BS_3_ are minimal; however, given that high molecular weight proteins are separated less on this type of gel (12%) even small fluctuations in migration distance may suggest a greater change in the observed molecular weight. Likewise, given that NHS reacts primarily with lysine residues, only two of which are available on membrane Band 3, we would expect the overall change in molecular weight to be small for the low molecular weight polymers designed in this study. Nonetheless, we believe that repeating these experiments under non-reducing conditions (i.e., to evaluate the interaction of NHS-polymers with membrane protein complexes), with polymer of higher molecular weight, and/or with a stronger cross-linking agent like diamide [56] would provide additional information and relevance in future experiments. Overall, the data presented in this study as a proof-of-concept investigation may suggest that Band 3 is a primary target of NHS-polymer binding and that binding may preclude membrane changes that precede clearance mechanisms. Membrane stabilization by NHS-pDMAA-Cy3 and its biocompatible nature were further supported by examining PS exposure in the outer membrane leaflet. Up to one hour following modification, no untoward effects on phospholipid dynamics in NHS-pDMAA-Cy3-modified erythrocytes were observed under physiological conditions (i.e, isotonic PBS, pH 7.4) (Fig 6A). Taken together, these data suggest that ATRP-synthesize, cell surface-reactive polymers do not promote membrane skeleton instability and may serve to enhance the circulation half-life of erythrocytes; a specific benefit in the context of their utility as biological drug carriers.

### Effects of Membrane Engineering on Human Erythrocyte Membrane Stabilization under Physiological and Oxidizing Conditions

In the circulation, erythrocytes encounter both endogenous and exogenous sources of reactive oxygen species (ROS) [57]. The majority of ROS species encountered by the erythrocyte are neutralized by cytosolic antioxidants; however, these intrinsic mechanisms become more limited in their function as blood flows through the microcirculation and hemoglobin becomes partially oxygenated [57]. Further, erythrocytes in the microcirculation make contact with the vasculature, where they are exposed to ROS release from neutrophils, macrophages, and endothelial cells [57].

The model of membrane destabilization utilized in this study relies on the generation of hydroxyl radicals through the breakdown of hydrogen peroxide via the Fenton reaction [49]. Hydroxyl radicals are neutralized by reacting with oxidizable entities nearby, inducing oxidative damage [49]. Phosphatidylserine (PS) becomes more numerous in the outer membrane leaflet of erythrocytes as they age, are subject to oxidative stress, and undergo lipid peroxidation [14–16] making it a reliable marker of membrane damage. Thus, the measurement of PS externalization was a useful marker for monitoring oxidative damage to the membranes of erythrocytes modified in this study.

We first noted reductions in PS externalization in hRBCs surface modified with NHS-pDMAA-Cy3 polymers despite incubation in oxidizing conditions up to one hour (Fig 6B). Subsequent experiments aimed to determine whether surface polymer modification had a temporal effect on erythrocyte oxidation (Figs 6C–6F and 8). Qualitative observations suggested that the protection afforded by surface modification were greatest soon following modification and declined to unmodified hRBC levels over 2 hours. Flow cytometry was used to more accurately determine oxidation levels in hRBCs with or without antioxidant-containing polymers under oxidative conditions. As such, NHS-pDMAA polymers were synthesized to contain the stable nitroxide radical, TEMPO˙ (Fig 7). The cell-permeable, stable nitroxide radical TEMPO˙ (2,2,6,6-tetramethylpiperidin-1-yl)oxyl) and its hydroxylated form, Tempol (4-hydroxy-TEMPO), have illustrated significant antioxidant effects, including the neutralization of hydroxyl radicals [24–27].

The cytoprotective benefit of NHS-pDMAA-Cy3 surface modification in hRBCs exposed to oxidizing conditions was short-lived, although significant at an early time point (Fig 8). Surface modification of erythrocytes with TEMPO-containing polymers extended cytoprotection up to one hour, with significant or nearly-significant results (Fig 8).

Nitroxides do not remain unchained in biological systems. There are multiple mechanisms that result in the interconversion of the parent amine [24, 58]. Thus, TEMPO may be active in a variety of states in our experimental systems, providing the direct neutralization of hydroxyl radicals, the neutralization of secondary radicals, or the formation of a non-radical TEMPO adduct. In other studies, the benefits of TEMPO *in vitro* were shown to be concentration dependent; 100 μM TEMPO completely protected against photo-oxidative losses in cell viability in a murine macrophage model of photo-oxidative stress [24], while 10 mM was required to protect primary thymocytes from extracellular oxidative stress [26]. Overall, membrane engineering of human erythrocytes with NHS-pDMAA-TEMPO˙ polymers demonstrated oxidative protection in the studied *in vitro* system. Importantly, no cytotoxicity for hRBCs modified with NHS-pDMAA-TEMPO˙ was observed in this study. The precise mechanisms by which these polymers quench a variety of oxidative species and otherwise stabilize the erythrocyte membrane to effectively extend circulation half-life warrants further investigation. While the measures reported here were indirect assessments, they provided the first substantial evidence of the potential antioxidant and membrane stabilizing nature of these ATRP-synthesized polymers. We intend to utilize specific methodologies in the future to further characterize these traits such as electron paramagnetic resonance (EPR) spectroscopy and reveal their biomedical and clinical potential [59, 60]. Our findings may be applicable to the oxidative damage of other cell types, as the erythrocyte membrane is a suitable biomembrane model [3].

We have already envisioned the utility of hRBC membrane-reactive polymers for other projects. Autologous hRBCs could be easily surface-modified with polymers *ex vivo*, eliminating complex and damaging methods associated with payload encapsulation. Human RBCs carrying both drug-polymer and antibody-polymer combinations could be used to target therapeutic payloads to the endothelium, smooth muscle cells, or various immune cells [61]. Additionally, polymers carrying specific antibodies could be used to target therapeutic payloads to circulating cancer cells or to enhance their removal from the circulation [62].

The polymers synthesized in this study may have profound effects and utility in other experimental models. Modification of additional cell types or the production of various antioxidant-carrying membrane protein-reactive polymers may be of interest to research groups developing ophthalmic or oral drug delivery systems. Indeed, carbomer 934P and hyaluronic acid, two common tear substitutes, were found to by cytoprotective for conjuctival cells exposed to oxidative stress [63] and hydrogels containing the antioxidant gallic acid functionalized to a gelatin copolymer demonstrated a cytoprotective effect of the carrier material in an animal model of glaucoma [64]. Additionally, modification of cell surfaces with antioxidant-carrying membrane-reactive polymers could protect implanted cells in inflammatory environments marked by elevated macrophage and neutrophils ROS generation. Support for this comes from a recent study demonstrating the potential for antioxidants like proanthocyanidin to protect human gingivial fibroblasts from increasing intracellular ROS [65]. Thus, the work presented herein may benefit several other research endeavors in tissues and cell types both within and outside of the circulatory system.

## Conclusion

Using ATRP methods, we were able to create biocompatible, surface reactive polymers that were homogenously retained on the periphery of modified erythrocytes for at least 24 hours. Moreover, the targeted functionalization of Band 3 protein in the erythrocyte membrane stabilized its monomeric form preventing aggregation in the presence of a surface impermeable crosslinking reagent. Moreover, erythrocyte membrane engineering with a free radical scavenging polymer, NHS-pDMAA-TEMPO˙, provided additional protection of surface modified erythrocytes in an *in vitro* model of oxidative stress. The hypothesized stabilization of membrane dynamics and the neutralization of hydroxyl radicals or hydroxyl radical-derived species may underlie the cytoprotective effects of nitroxide-containing polymers synthesized in this study. The capacity to neutralize oxidative species is an important consideration for the use of cell-based drug delivery methods that are likely to encounter areas of imbalanced oxidative stress that would compromise therapeutic efficacy.

## Supporting Information

S1 File Supplementary figures: Figure A: Synthesis of NHS-pDMAA-Cy3, Figure B: ^1^H NMR of P1 in *D*_2_O, Figure C: ^1^H NMR of HO-pDMAA-Cy3 in *D*_2_O, Figure D: ^1^H NMR of NHS-pDMAA-Cy3 in *D*_2_O, Figure E: Synthesis of NHS-pDMAA-TEMPO, Figure F: ^1^H NMR of P2 in *D*_2_O, Figure G: ^1^H NMR of P3 in *D*_2_O, Figure H: Additional Gels. (PDF)
