# Supplementary material for: The Effect of Covalently-Attached ATRP-Synthesized Polymers on Membrane Stability and Cytoprotection in Human Erythrocytes
Source: PLoS One. 2016 Jun 22;11(6):e0157641. doi: 10.1371/journal.pone.0157641 (PMC4917246; doi:10.1371/journal.pone.0157641)
Supplement: S1 File — (PDF) [file pone.0157641.s001.pdf]

Supporting Materials

Materials and Methods

N-hydroxysuccinimide (NHS), *N*-(3-dimethylaminopropyl)-*N*'-ethylcarbodiimide hydrochloride (EDC·HCl), 4-Amino-2,2,6,6-tetramethylpiperidine-1-oxyl (amino-TEMPO) copper (II) chloride ($CuCl_2$), and dimethyl sulfoxide (DMSO) were purchased from Sigma-Aldrich (St Louis, MO). *N,N*-dimethylacrylamide (DMAA; Sigma-Aldrich) was distilled in the presence of calcium hydride under vacuum. DisulfoCy3-NHS was purchased from Cyandye (Sunny Isles Beach, FL). ATRP initiators, *N*-2-chloropropionyl-β-alanine and *N*-(2-chloropropionyl)-4-butyric acid benzyl ester, were synthesized according to procedures reported by references [1] and [2] respectively. *N*-acryloyl-6-aminohexanoic acid (CAm) was synthesized according to procedure reported by reference [2]. Tris[2-(dimethylamino)ethyl]amine (Me_6TREN) was prepared according to a procedure reported by reference [3].

Measurements

^{1}H NMR spectra (S2-S4 Figs; S6 and S7 Figs) were recorded on a spectrometer (300 MHz) in the NMR facility located in Center for Molecular Analysis, Carnegie Mellon University, with Deuterium oxide (D_2O). Routine FT-IR spectra were obtained with an ATI Mattson Infinity Series FT-IR spectrometer. UV−vis spectra were obtained using a UV−vis spectrometer (Lambda 2, PerkinElmer).Number average molecular weights (M_n) and the polydispersity index (M_w/M_n) were estimated by gel permeation chromatography (GPC) on a Water 2695 Series with a data processor, equipped with three columns (Waters Ultrahydrogel Linier, 500 and 250), using 80 vol % of 100 mM sodium phosphate buffer (pH 9.0) with 20 vol % acetonitrile as an eluent at a flow rate 1.0 mL/min, with detection by a refractive index (RI) detector. Polyethylene glycol standards were used for calibration. 2,4,6-trintrobenzene sulfonic acid (TNBS) assay was carried out to determine amine groups of the polymer. TNBS solution (250 μL, 0.1 % TNBS in

100 mM sodium phosphate buffer (pH 8.5)) was added into the polymer solution (500 µL, 1.0 mg / mL in 100 mM sodium phosphate buffer (pH 8.5)), then incubated at 37 °C for 2h. After adding water (375 µL) to the incubated solution, the absorbance of the solution at 345 nm was measured by UV-vis spectrometer using PMMA cuvette. 4-Amino-2,2,6,6-tetramethylpiperidine-1-oxyl (amino-TEMPO) was used for calibration.

Synthesis of P1

N-2-chlorolpropionyl-β-alanine as an ATRP initiator (18 mg, 0.1 mmol), DMAA (535 µL, 5 mmol), sodium ascorbate (40 mg, 0.2 mmol) in deionized water (20 mL) were placed in a polymerization flask. The polymerization solution was charged with nitrogen gas for 30 min and then nitrogen gas charged solution of Me_6TREN (40 µL, 0.15 mmol) and $CuCl_2$ (16 mg, 0.12 mmol) in deionized water (1 mL) was added under nitrogen gas flow. The polymerization was carried out at 4 °C for 4h, then nitrogen gas charged amino-TEMPO (4-amino-2,2,6,6-tetramethylpiperidine-1-oxyl, 86 mg, 0.5 mmol) in deionized water (1 mL) was added under nitrogen gas flow. The solution was stirred at 4 °C overnight. The resulting mixture was isolated by dialysis with Mwco 1000 Da dialysis tube (Spectra/Por®, Spectrum Laboratories Inc., Rancho Dominguez, CA) in deionized water overnight, and then the polymer was lyophilized. Number average molecular weight (M_n) 6.5 kDa and the distributions (M_w/M_n) 1.23 by GPC. IR (KBr) 3454, 2938, 1624, 1500, 1405, 1359, 1255, 1149, 1058 and 630 cm^{-1}. 78 mol% of polymer end could be converted to amine group by chain termination of the polymerization using amino-TEMPO.

Preparation of HO-pDMAA-Cy3

Sulfo-Cyanine3 NHS ester (30 mg, 41 µmol) was added in solution of the polymer (260 mg, 31 µmol of $–NH_2$ end group) and trimethylamine (11 µL, 80 µmol) in DMSO (5 mL), and then stirred

at room temperature for 2 h. The polymer, **HO-pDMAA-Cy3**, was isolated by dialysis using an Mwco 1,000 Da dialysis tube in deionized water overnight, and then the polymer was lyophilized. Estimated molecular weight (M_n) 7.0 kDa by ^{1}H NMR spectrum. IR (KBr) 3434, 2935, 1627, 1561, 1499, 1451, 1406, 1360, 1256, 1147, 1111, 1059, 1026, 931 and 630 cm^{-1}.

Preparation of NHS-pDMAA-Cy3

EDC·HCl (46 mg, 240 µmol) and NHS (28 mg, 240 µmol) were then added to solution of the HOOC-pDMAA-Cy3 (170 mg, 24 µmol of -COOH end group) in deionized water (10 mL) at 0 °C and stirred at room temperature for 30 min. The polymer, **NHS-pDMAA-Cy3**, were isolated by dialysis using an Mwco 1,000 Da dialysis tube in the refrigerator and then lyophilized. Estimated molecular weight (M_n) 7.1 kDa by ^{1}H NMR spectrum. IR (KBr) 3454, 2937, 1811, 1773, 1734, 1627, 1561, 1502, 1458, 1404, 1359, 1255, 1147, 1096, 1059, 1026, 931 and 632 cm^{-1}.

Preparation of P2

Carboxyl group protected ATRP initiator (*N*-(2-chloropropionyl)-4-butyric acid benzyl ester, 56 mg, 0.2 mmol), DMAA (1.03 mL, 10 mmol), sodium ascorbate (80 mg, 0.4 mmol) in ethanol (4 mL) and deionized water (16 mL) were placed in a polymerization flask. The polymerization solution was charged with nitrogen gas for 30 min and then nitrogen gas charged solution of Me_6TREN (60 µL, 0.22 mmol) and $CuCl_2$ (30 mg, 0.22 mmol) in deionized water (1 mL) was added under nitrogen gas flow. The polymerization was carried out at room temperature for 4 h, then nitrogen gas charged DMAA (618 µL, 6.0 mmol), *N*-acryloyl-6-aminohaxanoic acid, (CAm, 740 mg, 4.0 mmol) and sodium hydrogen carbonate (336 mg, 4.0 mmol) in deionized water (10 mL) was added under nitrogen gas flow. The solution was stirred at room temperature overnight. The resulting mixture was dialyzed by using an Mwco 1000 dialysis tube in deionized water overnight, and then the isolated polymer was lyophilized. Number average molecular

weight (M_n) 7.0 kDa and the distributions (M_w/M_n) 1.32 by GPC. IR (KBr) 3453, 2935, 2876, 1725, 1720, 1626, 1500, 1460, 1405, 1360, 1257, 1146, 1057 and 622 cm^{-1}. 4.9 of carboxyl groups per polymer chain were estimated by ^{1}H NMR spectrum.

Preparation of P3

EDC·HCl (191 mg, 1.0 mmol) and NHS (115 mg, 1.0 mmol) were added to a solution of the obtained polymer (450 mg, 0.1 mmol polymers i.e. ca. 0.5 mmol of COOH groups) in acetonitrile (10 mL) at 0 °C then stirred at room temperature for 30 min. Amino-TEMPO (214 mg, 1.25 mmol) was added to the reaction mixture and stirred at room temperature overnight. 1 N NaOH aq. (150 µL) was added to the mixture and stirred at room temperature for 2 h. The resulting polymer was lyophilized after isolated by dialysis using an Mwco 1000 dialysis tube in deionized water overnight. 3.1 TEMPO groups per polymer chain were estimated by ^{1}H NMR spectrum. Estimated molecular weight (M_n) 7.3 kDa by ^{1}H NMR spectrum. IR (KBr) 3453, 2935, 2875, 1626, 1500, 1460, 1405, 1360, 1256, 1148, 1057 and 631 cm^{-1}.

Preparation of NHS-pDMAA-TEMPO·

EDC·HCl (96 mg, 0.5 mmol) and NHS (58 mg, 0.5 mmol) were then added to a solution of polymer (**P3**) (300 mg, 0.05 mmol of COOH group) in deionized water (10 mL) and stirred at room temperature for 30 min. The final polymer (**NHS-pDMAA-TEMPO·**) was isolated by dialysis using an Mwco 1000 dialysis tube in the refrigerator and then lyophilized. Estimated molecular weight (M_n) 7.4 kDa by ^{1}H NMR spectrum. IR (KBr) 3435, 2936, 2881, 1811, 1773, 1734, 1627, 1502, 1459, 1435, 1404, 1359, 1255, 1208, 1147, 1057 and 633 cm^{-1}.

References

1. Cummings C, Murata H, Koepsel R, Russell AJ (2014) Dramatically increased pH and temperature stability of chymotrypsin using dual block polymer-based protein engineering. Biomacromolecules 15: 763-771.

2. D'Souza S, Murata H, Jose MV, Askarova S, Yantsen Y, et al. (2014) Engineering of cell membranes with a bisphosphonate-containing polymer using ATRP synthesis for bone targeting. Biomaterials 35: 9447-9458.

3. Ciampolini M, Nardi N (1966) Five-coordinated high-spin complexes of bivalent cobalt, nickel, and copper with tris(2-dimethylaminoethyl)amine. Inorg Chem 5: 41-44.

Supporting Materials Figures

Figure A: Synthesis of **NHS-pDMAA-Cy3**

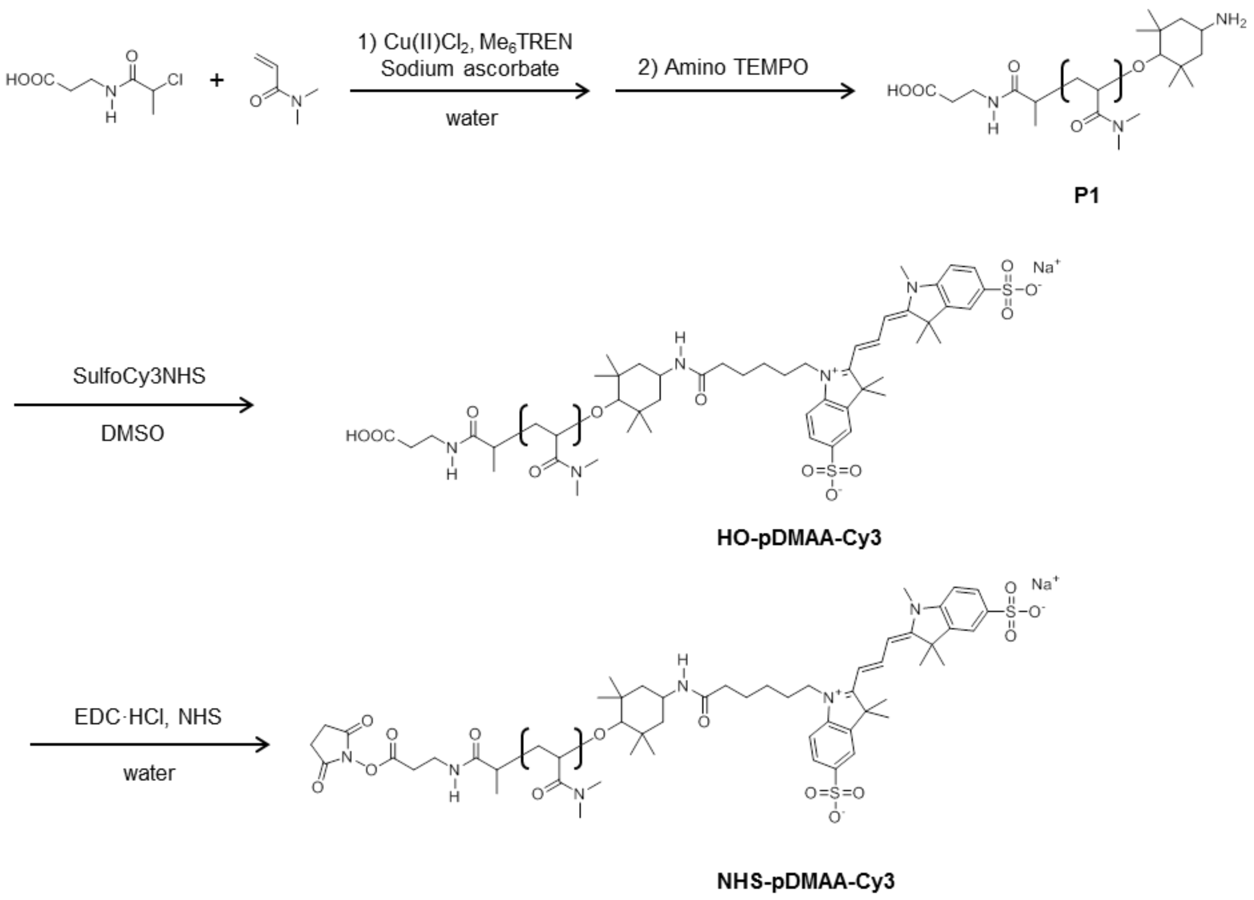

Figure B: ^{1}H NMR of **P1** in D_2O

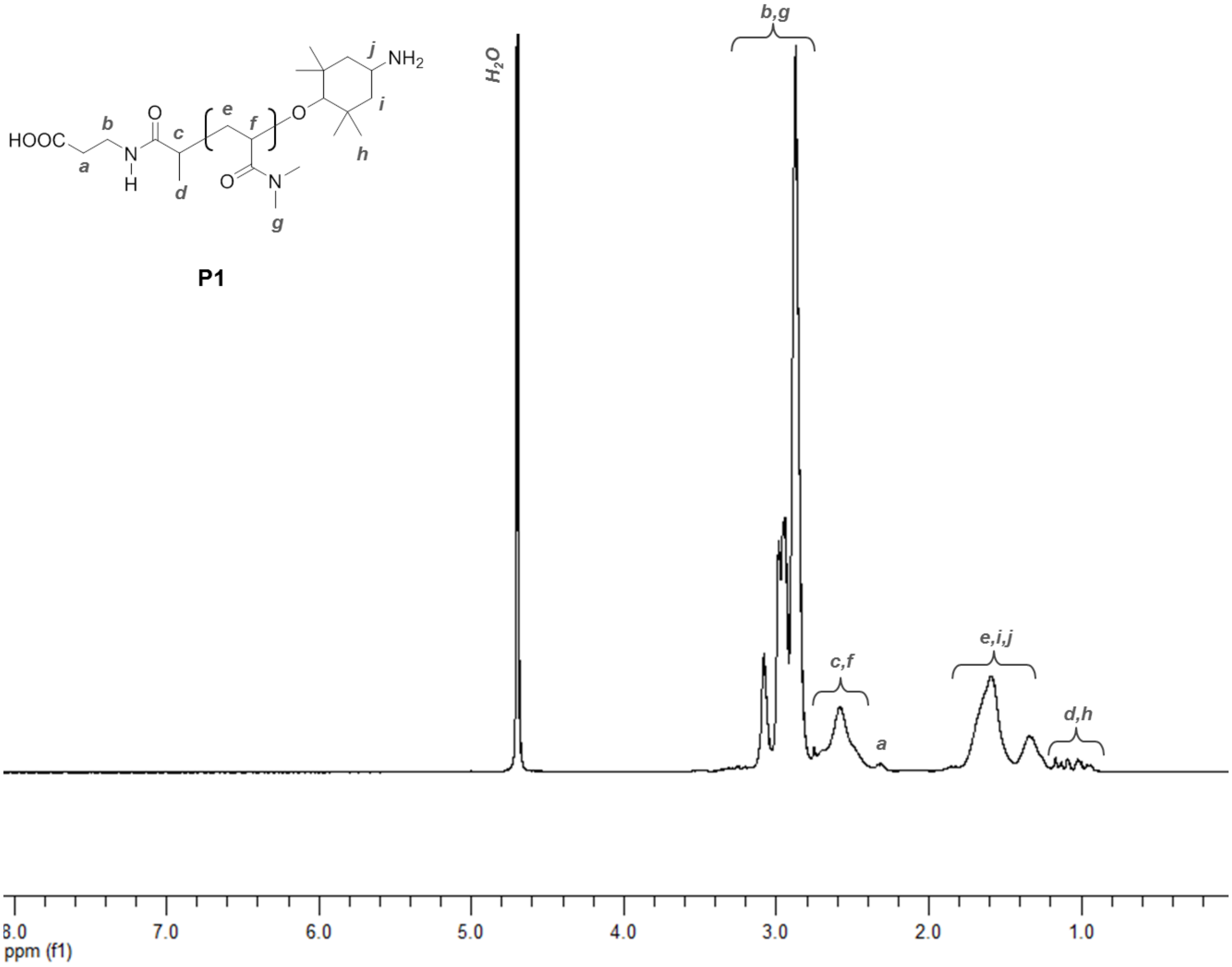

Figure C: ^{1}H NMR of **HO-pDMAA-Cy3** in D_2O

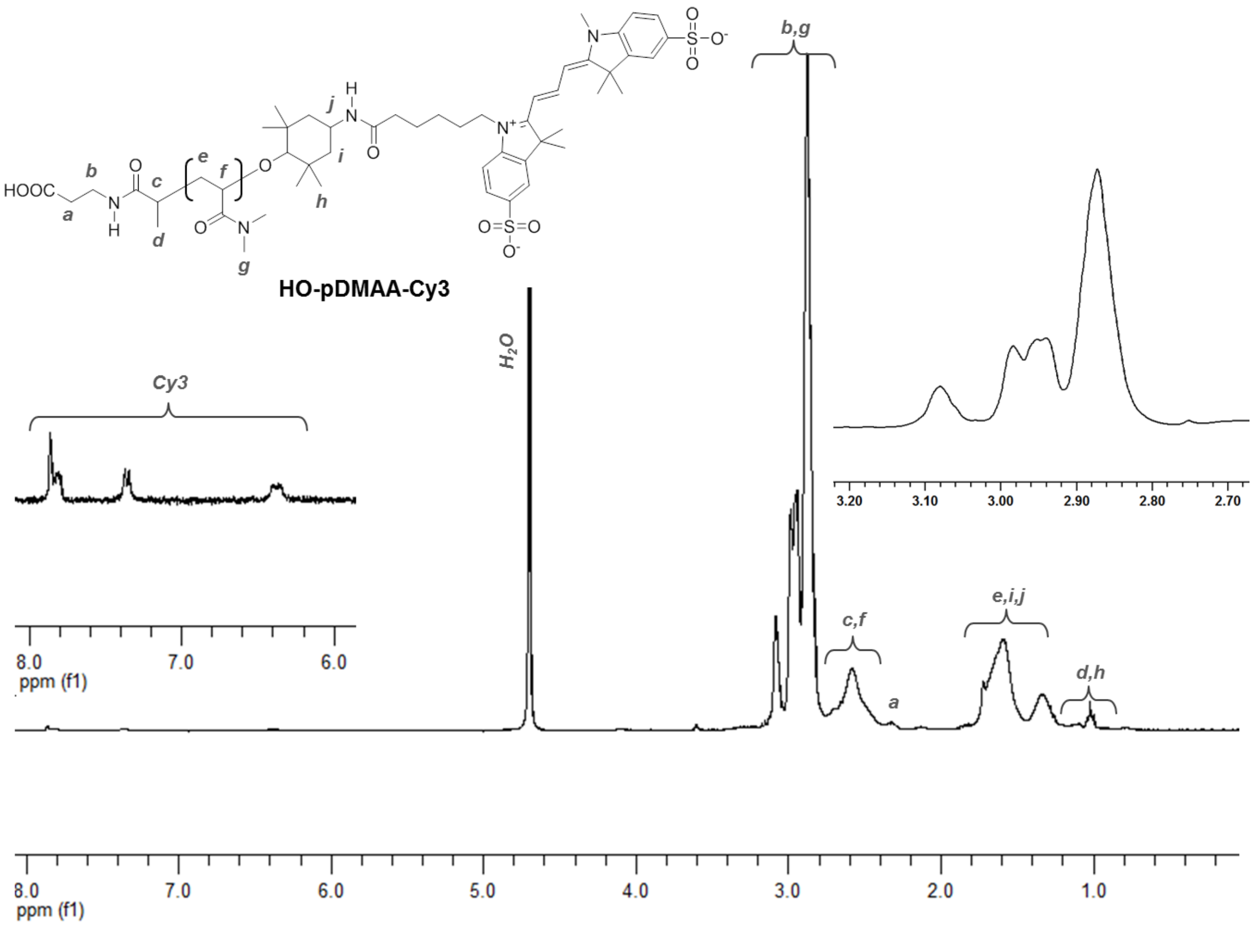

Figure D: ^{1}H NMR of **NHS-pDMAA-Cy3** in D_2O

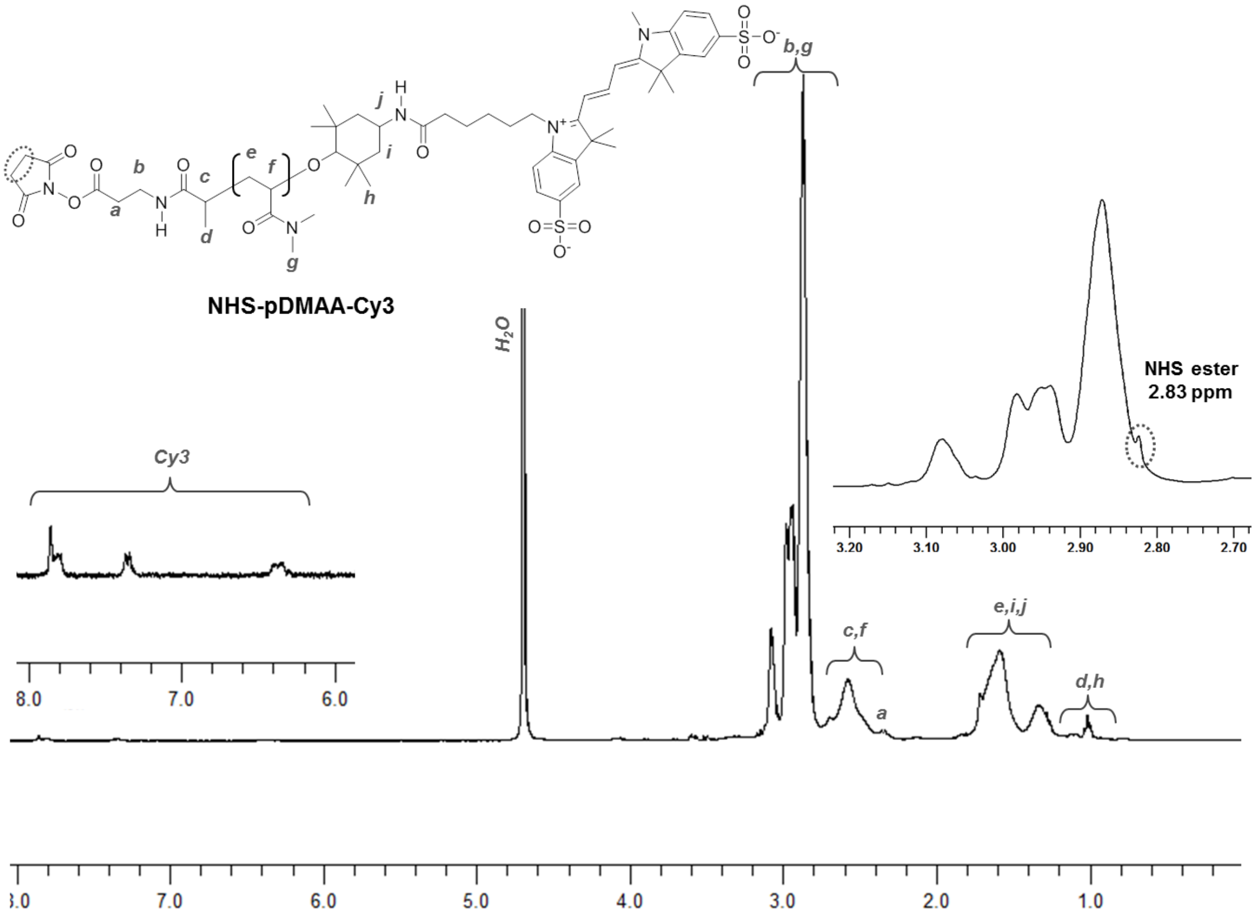

Figure E: Synthesis of **NHS-pDMAA-TEMPO·**

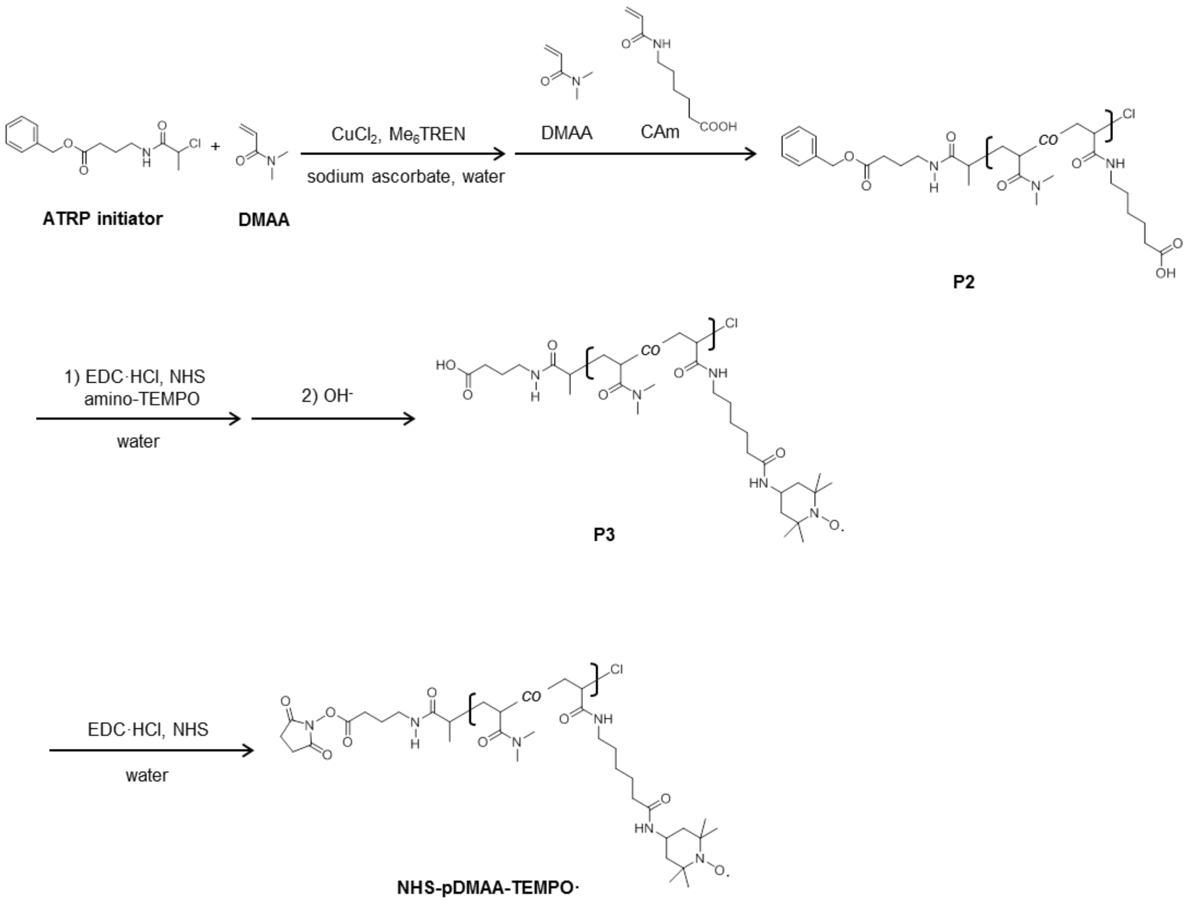

Figure F: ^{1}H NMR of **P2** in D_2O

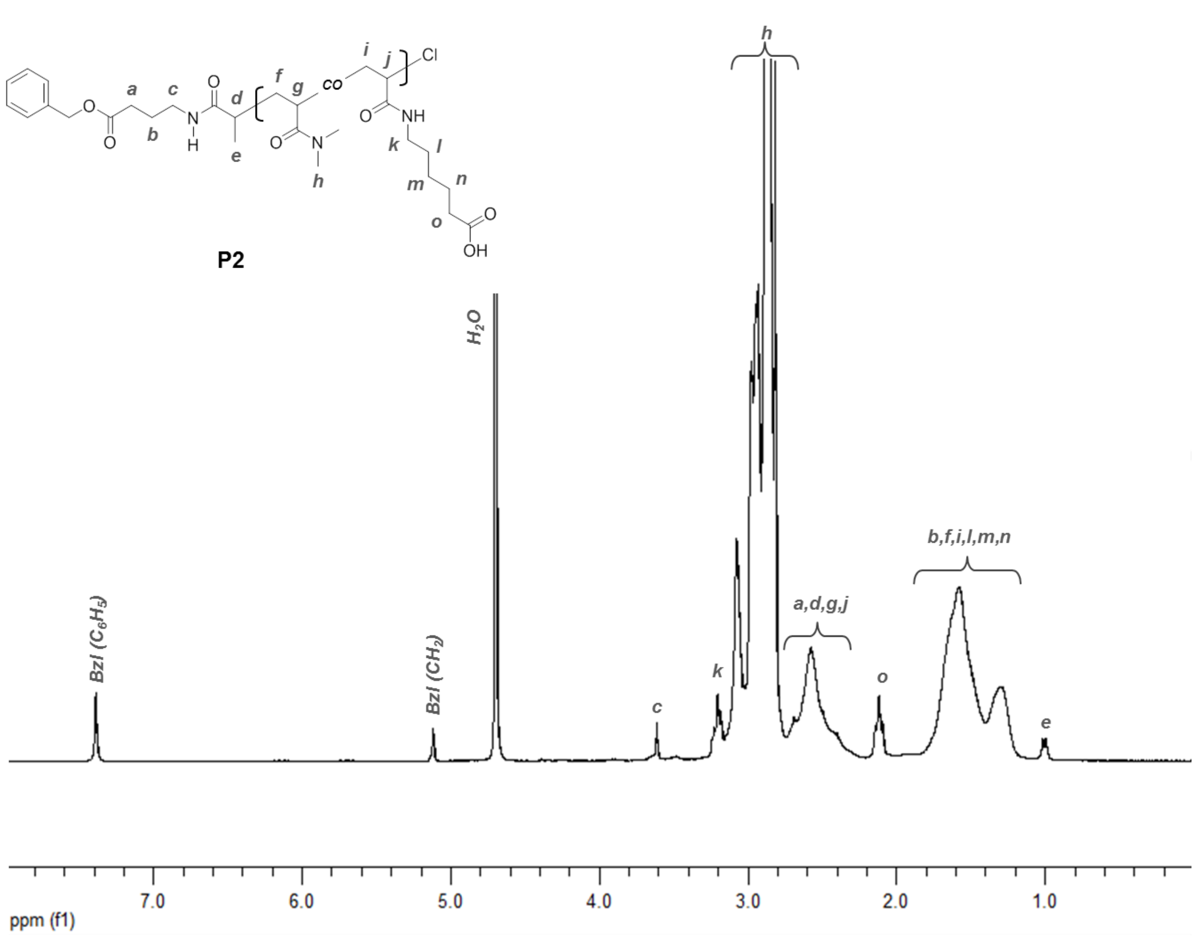

Figure G: ^{1}H NMR of **P3** in D_2O

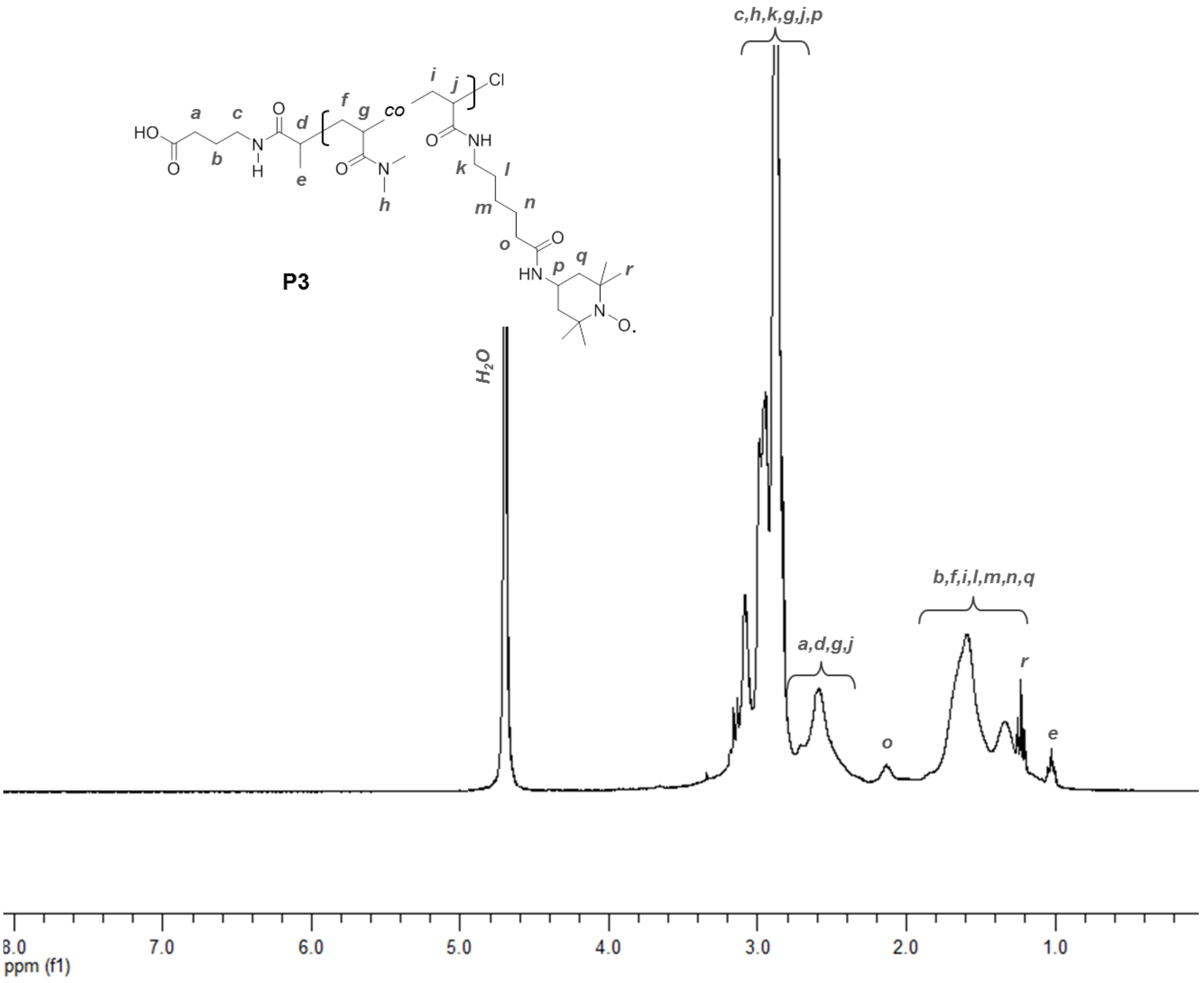

Figure H. Additional Gels

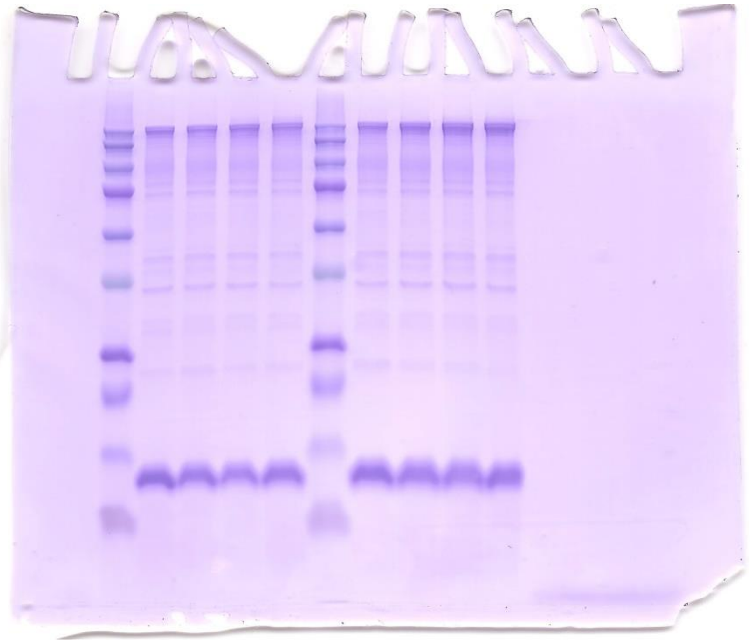

02252015 Trial 2 BS3 crosslinking of Band 3 in hRBC modified with NHS-Cy3

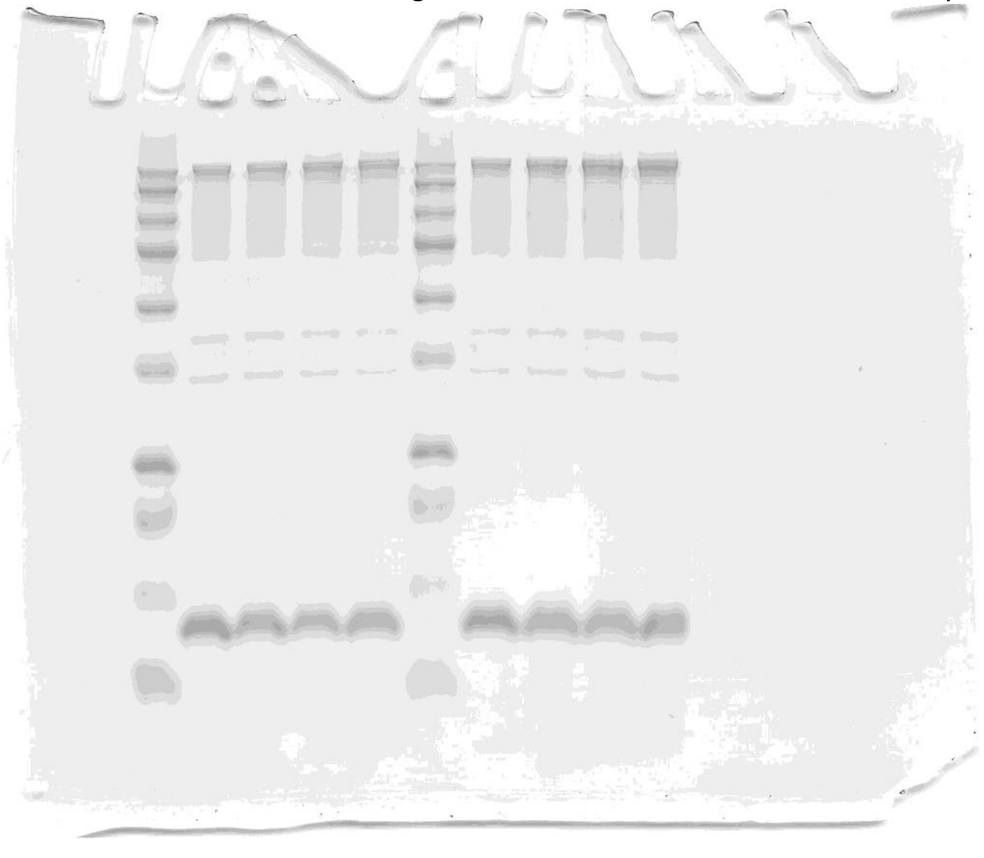

02252015 Trial 2 BS3 crosslinking of Band 3 in hRBC modified with NHS-Cy3 -Grey Scale

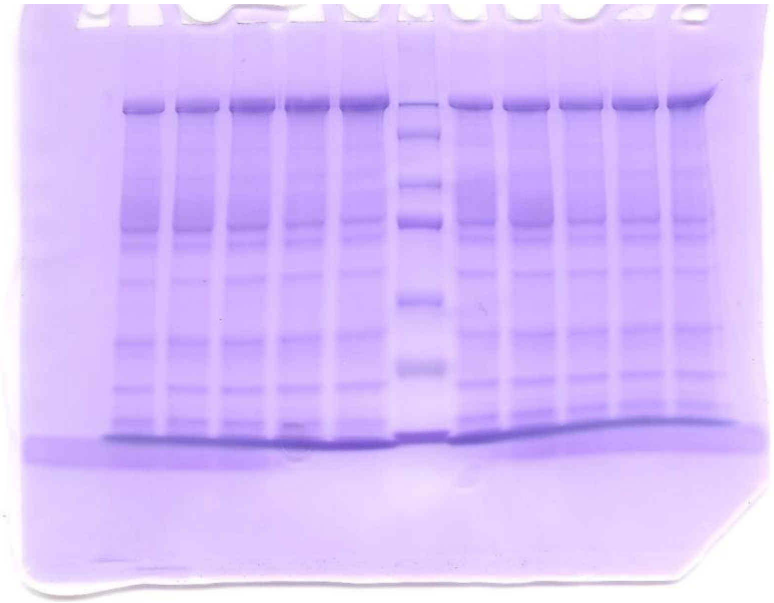

03132015 Trial 5 BS3 crosslinking of Band 3 in hRBC modified with NHS-Cy3

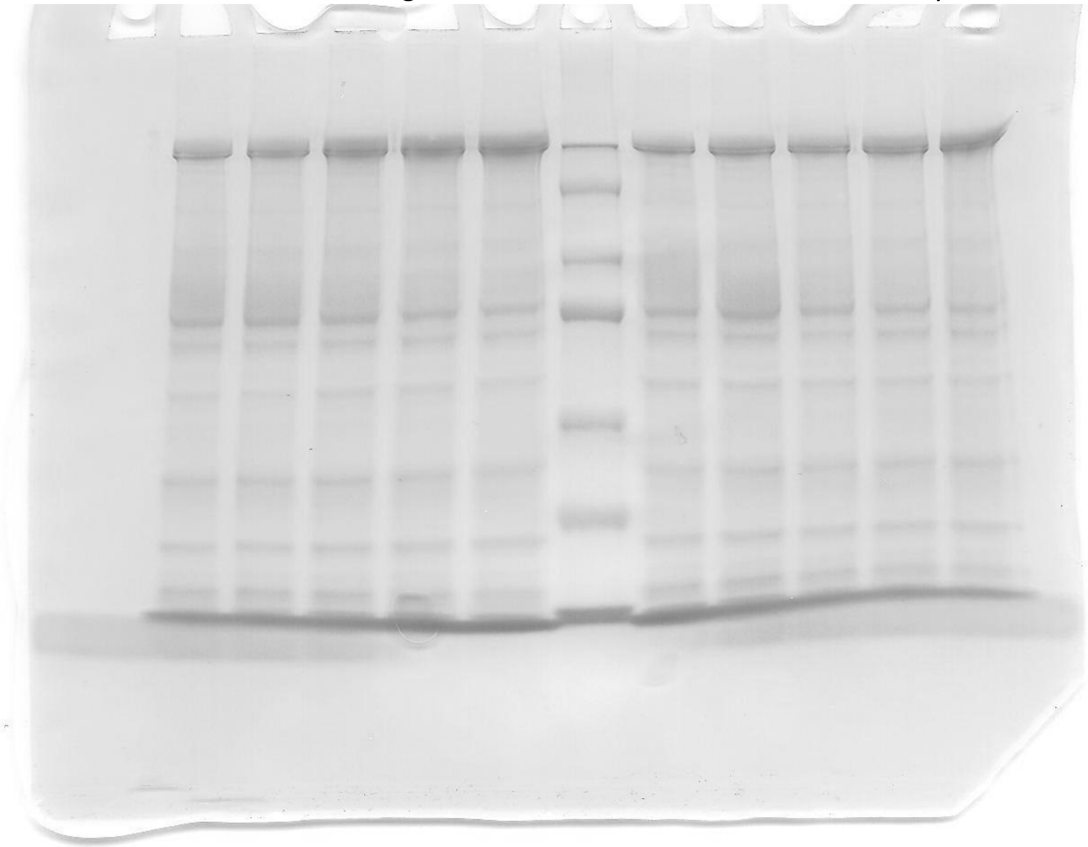

03132015 Trial 5 BS3 crosslinking of Band 3 in hRBC modified with NHS-Cy3- grey scale

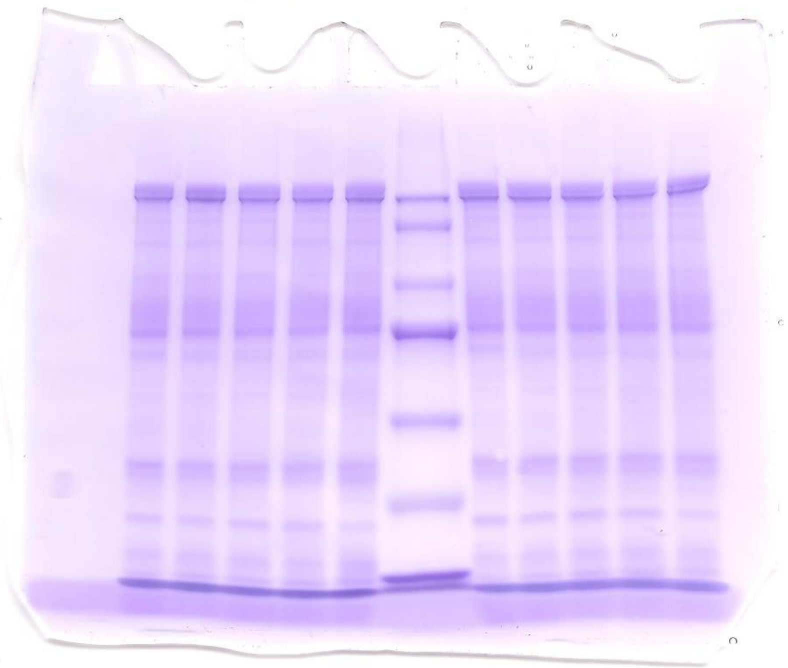

03192015 Trial 7 BS3 crosslinking of Band 3 in hRBC modified with NHS-Cy3

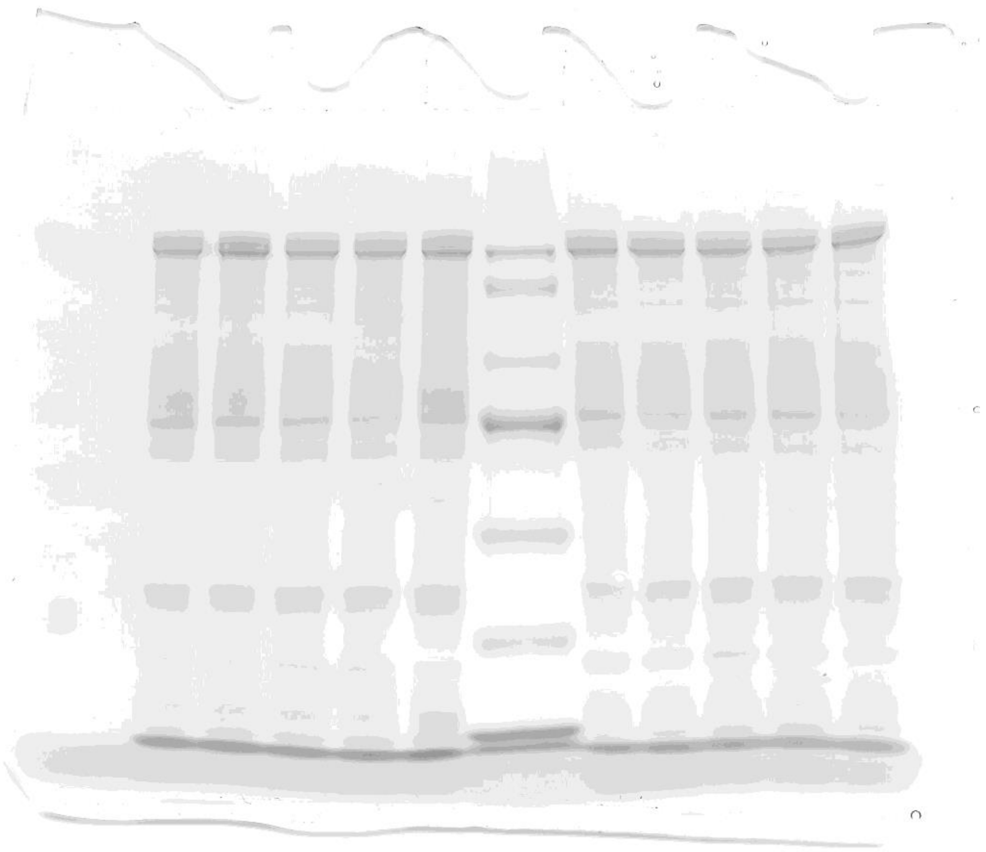

03192015 Trial 7 BS3 crosslinking of Band 3 in hRBC modified with NHS-Cy3 grey scale
